# Distribution of Patients at Risk for Complications Related to COVID-19 in the United States: Model Development Study

**DOI:** 10.2196/19606

**Published:** 2020-06-18

**Authors:** Renae Smith-Ray, Erin E Roberts, Devonee E Littleton, Tanya Singh, Thomas Sandberg, Michael Taitel

**Affiliations:** 1 Walgreens Center for Health and Wellbeing Research Health Analytics, Research, and Reporting Walgreen Co Deerfield, IL United States; 2 Healthcare Planning and Research Walgreen Co Deerfield, IL United States

**Keywords:** COVID-19, modeling, chronic conditions, older adults

## Abstract

**Background:**

Coronavirus disease (COVID-19) has spread exponentially across the United States. Older adults with underlying health conditions are at an especially high risk of developing life-threatening complications if infected. Most intensive care unit (ICU) admissions and non-ICU hospitalizations have been among patients with at least one underlying health condition.

**Objective:**

The aim of this study was to develop a model to estimate the risk status of the patients of a nationwide pharmacy chain in the United States, and to identify the geographic distribution of patients who have the highest risk of severe COVID-19 complications.

**Methods:**

A risk model was developed using a training test split approach to identify patients who are at high risk of developing serious complications from COVID-19. Adult patients (aged ≥18 years) were identified from the Walgreens pharmacy electronic data warehouse. Patients were considered eligible to contribute data to the model if they had at least one prescription filled at a Walgreens location between October 27, 2019, and March 25, 2020. Risk parameters included age, whether the patient is being treated for a serious or chronic condition, and urban density classification. Parameters were differentially weighted based on their association with severe complications, as reported in earlier cases. An at-risk rate per 1000 people was calculated at the county level, and ArcMap was used to depict the rate of patients at high risk for severe complications from COVID-19. Real-time COVID-19 cases captured by the Johns Hopkins University Center for Systems Science and Engineering (CSSE) were layered in the risk map to show where cases exist relative to the high-risk populations.

**Results:**

Of the 30,100,826 adults included in this study, the average age is 50 years, 15% have at least one specialty medication, and the average patient has 2 to 3 comorbidities. Nearly 28% of patients have the greatest risk score, and an additional 34.64% of patients are considered high-risk, with scores ranging from 8 to 10. Age accounts for 53% of a patient’s total risk, followed by the number of comorbidities (29%); inferred chronic obstructive pulmonary disease, hypertension, or diabetes (15%); and urban density classification (5%).

**Conclusions:**

This risk model utilizes data from approximately 10% of the US population. Currently, this is the most comprehensive US model to estimate and depict the county-level prognosis of COVID-19 infection. This study shows that there are counties across the United States whose residents are at high risk of developing severe complications from COVID-19. Our county-level risk estimates may be used alongside other data sets to improve the accuracy of anticipated health care resource needs. The interactive map can also aid in proactive planning and preparations among employers that are deemed critical, such as pharmacies and grocery stores, to prevent the spread of COVID-19 within their facilities.

## Introduction

The first case of coronavirus disease (COVID-19) was detected in the United States on January 20, 2020 [1]. The spread of the virus increased exponentially across the United States during the subsequent two months, with large outbreaks occurring in urban localities including New York City, the San Francisco Bay Area, Detroit, and New Orleans [2].

The Centers for Disease Control and Prevention (CDC) analyzed data from lab-confirmed COVID-19 cases in the United States from February 12 to March 28, 2020. This analysis found that older adults and individuals with underlying health conditions are at higher risk of developing life-threatening complications from COVID-19 [3]. Among COVID-19 patients, 38% had one or more underlying health conditions, and the rates of hospitalization among these patients was disproportionately high. The majority of intensive care unit (ICU) admissions (78%) and non-ICU hospitalizations (71%) were patients with at least one underlying health condition.

Efforts to reduce mortality due to COVID-19 should include identifying and protecting patients who have the highest risk of developing severe complications from the disease. The purpose of this study was to develop a risk model to estimate the risk status for patients of a nationwide pharmacy chain in the United States and to identify the geographic distribution of patients who have the highest risk of severe COVID-19 complications.

## Methods

### Data Inputs and Sources

#### Pharmacy Data

Adult patients (aged ≥18 years) were identified from the Walgreens electronic data warehouse. Patients were considered eligible to contribute data to the model if they had at least one prescription filled at a Walgreens location between October 27, 2019, and March 25, 2020. Eligible patients were assigned a risk score based on the sum of each patient’s risk parameters including the following: an inferred diagnosis of a serious chronic condition based on a prescription fill within this period for certain specialty medications (Multimedia Appendix 1), an inferred diagnosis of a chronic condition that is deemed to put the patient at high risk of severe COVID-19 complications based on a prescription fill to treat these conditions (Multimedia Appendix 2), prescription fills which infer diagnosis of other chronic conditions, age, and urban density classification. Ethical approval was received from the Advarra Institutional Review Board (protocol number 35300).

Our team assigned a risk value to each parameter based on findings from recent COVID-19 studies [3,4]. The risk score algorithm weighted parameters based on their association with complications from COVID-19 infection, such as hospitalization and death. Parameters shown to be associated with the greatest risk of severe COVID-19 complications were assigned the highest value possible, regardless of the presence of other risk factors. The highest risk parameters included a prescription fill within the study period for one of the high-risk specialty medications and being aged 80 years and above.

Prescription fills to treat high-risk chronic conditions and other chronic conditions not deemed high-risk were assigned a value based on hazard ratios published in the European Respiratory Journal [5]. Patients with specific underlying health conditions are at high risk of developing severe complications from COVID-19 [3]. The risk score for patients with chronic lung disease, diabetes mellitus, and cardiovascular disease was weighted higher than the risk for patients being treated for other chronic conditions that do not fall into one of these three disease states. Baseline risk is determined by the number of medications the patient is on, and whether that medication is for treatment of any chronic condition. Patients treated with medication for one or more of the three high-risk conditions in addition to being treated with additional chronic condition medications received a cumulative value for each category. For instance, a patient being treated for chronic lung disease, diabetes mellitus, and one additional high-risk maintenance medication would receive the following values for these conditions: 2.681 + 1.586 + 2.592 = 6.459.

Compounding evidence shows that the risk of developing severe complications from COVID-19 increases exponentially with age; therefore, the risk score was weighted more heavily for older patients. Observational evidence shows that the spread of COVID-19 occurs most rapidly in urban areas. For this reason, we weighted patients who live in densely populated urban areas with the greatest risk, followed by those in less dense urban, suburban, and rural settings. Counties categorized as rural contain a population density of <400 people per square mile, suburban encompasses population density between 400 and 5000 people per square mile, less dense urban includes counties with 5000 to 12,500 people per square mile, and urban encompasses population density over 12,500 people per square mile. Population data were acquired from Popstats 2019 (Syergos Technologies Inc).

The risk model was developed using a training test split approach. The model was tested and validated using data for patients residing in one state (Georgia), and then applied to the full United States study cohort. Once cumulative risk values were calculated for each patient, the values were transformed to a maximum risk score of 10 to aid with interpretation using the following formula:


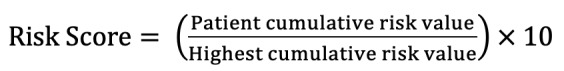


#### COVID-19 Surveillance Data

Real-time data of COVID-19 cases captured by the Johns Hopkins University Center for Systems Science and Engineering (CSSE) [2] was layered in the risk map to show where cases exist relative to the populations identified as being at high risk of severe complications from COVID-19.

### Model Validation

The model was compared with current trends in COVID-19 cases. Without the availability of confirmed cases, the predictive value of this model is unknown [6].

### Mapping

ArcMap (Esri) was used to depict the presence of patients identified as being at high risk for severe complications from COVID-19 and real-time COVID-19 cases. The at-risk rate per 1000 people is provided at the county level. County populations of fewer than 100 residents or fewer than 10 patients were excluded from the data set. The combined view shows where cases exist relative to the populations identified as high-risk. Additionally, testing locations, Walgreens store, and clinic locations are seen with a zoomed in view. The ArcGIS Online platform (Esri) was used to distribute this map publicly beginning April 16, 2020.

## Results

The study included 30,100,826 adults filling at least one specialty or maintenance medication during the study period. Table 1 shows the model inputs and parameters. Using a training test split approach, the model was tested and validated on 623,972 patients residing in Georgia and applied to the full US study cohort (N=30,100,826).

The average age of patients is 50 years, and the average patient has 2 to 3 comorbidities. Nearly 28% (8,285,408) of patients have the greatest risk score, and 10,426,683 (34.64%) of patients are considered high-risk (a score of at least 8; Table 2). The mean risk score before standardization is 7.81. Age accounts for 52% (4.04) of a patient’s total risk, followed by the number of inferred comorbidities (29%; 2.23); inferred chronic obstructive pulmonary disease, hypertension, or diabetes (15%; 1.21); and the urban density classification (5%; 0.38).

The risk assigned is most heavily weighted for adults aged ≥80 years (maximum value assigned), followed by adults aged 65 to 79 years [(7 + age/100)], 50 to 64 years [(1 + age/100)^3^], and 18 to 49 years [(1 + age/100)^2^].

**Table 1:** Model inputs and values.

Risk factor		Risk value
**Baseline risk**		
	Maintenance medications for a non–high-risk chronic condition	1.789
	Maintenance medications for a high-risk chronic condition	2.289
	Maintenance medications (≥2)	2.592
**Known disease states for risk**		
	Specific specialty medications	Maximum
	Chronic lung disease medications	2.681
	Diabetes mellitus medications	1.586
	Cardiovascular disease medications	1.575
**Age-related risk**		
	Age of 18-49 years	(1 + age/100)^2^
	Age of 50-64 years	(1 + age/100)^3^
	Age of 65-79 years	(7 + age/100)
	Age of ≥80 years	Maximum
**Urban density classification risk**		
	Urban	1
	Less dense urban	0.75
	Suburban	0.5
	Rural	0

**Table 2:** Risk category summary (N=30,100,826).

Risk category	Patients, n (%)
≤1	765,867 (2.54)
≤3	6,598,930 (21.92)
≤4	4,594,748 (15.26)
≤5	2,999,058 (9.96)
≤6	2,374,706 (7.89)
≤7	2,340,834 (7.78)
≤8	1,679,440 (5.58)
≤9	447,802 (1.49)
<10	14,033 (0.05)
10	8,285,408 (27.53)

**Figure 1:**
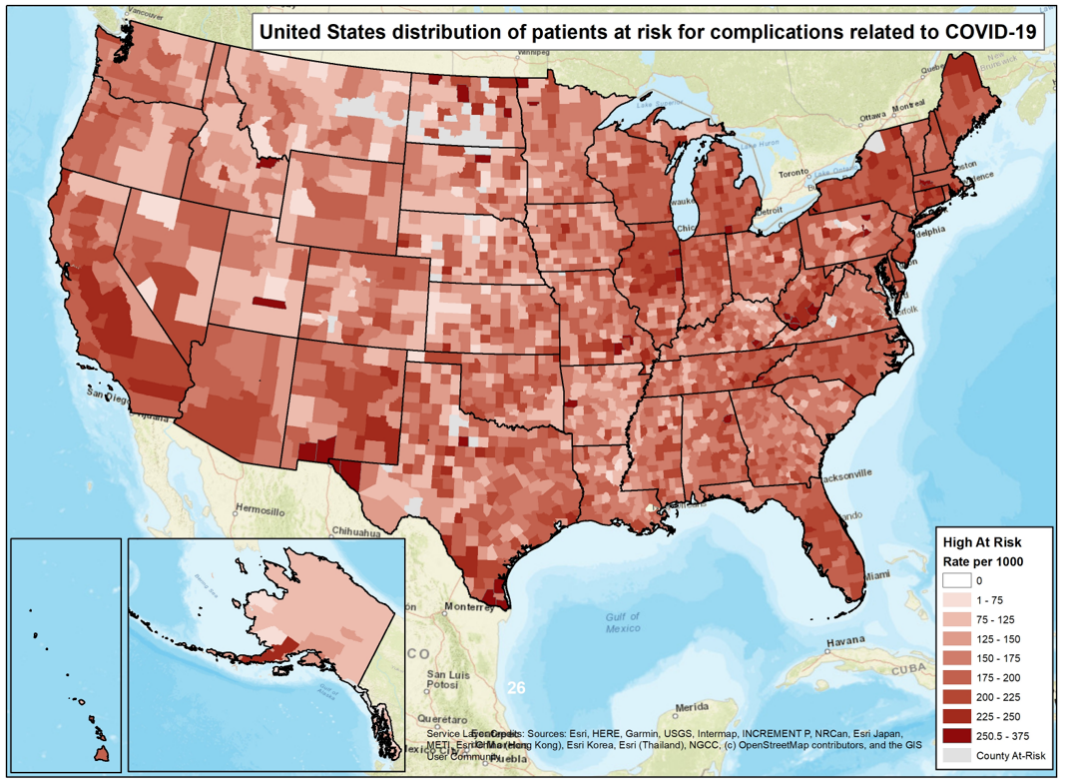
Distribution of patients at risk for complications related to coronavirus disease (COVID-19) in the United States.

Patient addresses were used to depict the distribution of risk status across the United States. These data were then compiled to depict a county-level risk status for each county for which we had sufficient data. A county-level at-risk rate was calculated per 1000 residents. The highest county-level risk category ranged from 265.1 to 375.0 high-risk residents per 1000. Furthermore, 8 risk ranges were assembled and color coded onto a county-level US map (Figure 1). The real-time Johns Hopkins University CSSE COVID-19 cases data are layered on top of the county-level risk status to facilitate a visual depiction of the presence of cases in relation to the county-level risk of residents at risk of suffering severe complications from COVID-19 [2]. At the time of publication, the map depicts numerous counties, principally in less densely populated regions of the United States that have a high rate of vulnerable residents but have not yet had large numbers of COVID-19 cases. The interactive map depicting the US distribution of patients at risk for complications related to COVID-19 is publicly available for viewing [7]. The county-level risk rates are recalculated and refreshed weekly, whereas the Johns Hopkins University CSSE case numbers are uploaded in real time.

## Discussion

### Overview

This study shows that there are counties across the United States whose residents are at high risk of developing severe complications from COVID-19; many of these counties had not yet recorded many COVID-19 cases when the interactive map was released. Although transmission rates may differ among rural and urban areas, it is often the case that residents of rural counties have higher risk statuses and less access to health care resources. If disease transmission becomes rampant in a rural county with a high risk status, health care resources may become depleted quickly if a disproportionate number of its residents experience severe complications from the disease.

This risk model utilizes data from approximately 10% of the US population. At the time of publication, this is the most comprehensive US model to depict county-level prognosis of COVID-19 infection [8]. DeCaprio et al [9] modeled rates of COVID-19–related pneumonia and hospital admission using 1.5 million records from Medicare claims data from 2015 to 2016. Unlike medical claims data, our pharmacy claims data is accessible at a near real-time rate, which likely improves the precision of the model. Moreover, our data includes US adults aged 18 years and above, making our population estimates broader and more generalizable.

With the core data, Walgreens was able to implement proactive community outreach by pharmacists who offered home delivery to high-risk patients to ensure they had a sufficient supply of their medications without having to leave their homes. The pharmacists also inquired about patients’ wellbeing during the pandemic and shelter-in-place orders, and they referred patients to community services as needed. Additionally, by publicly sharing deidentified county-level risk distributions, Walgreens and other organizations are able to plan and respond as COVID-19 begins to spread to areas that previously experienced little impact.

More importantly, our interactive map will serve to inform public officials and health care leaders of where there are highly vulnerable pockets of the population so that they may proactively prepare for the possibility of a disproportionately high number of patients with severe complications due to COVID-19. Many of these high-risk populations are in rural areas that have limited access to advanced health care services such as a hospital with respirators. Other maps have depicted the current availability of health care resources, such as ICU beds, compared to the amount that will be required in the event of a regional COVID-19 outbreak [10]. Our county-level risk estimates may be used alongside data sets such as that produced by Moghadas et al [10] to improve the accuracy of anticipated health care resource needs.

Our interactive map will also aid in proactive planning and preparations among employers that are deemed critical, such as pharmacies and grocery stores, to prevent the spread of COVID-19 within their facilities. At the time of publication, the interactive map showed that it is relatively uncommon to see a county with a low rate of patients at risk for complications related to COVID-19, but a high rate of COVID-19 cases. This may be evidence of the differential presentation of SARS-CoV-2 (severe acute respiratory syndrome coronavirus 2) in individuals who are younger and have few comorbidities as compared to their counterparts.

### Limitations

There is potential bias in the data source as it only includes Americans who have access to health care and can afford to purchase medication. The model would likely be strengthened if it represented less-advantaged individuals who are uninsured or underinsured, as well as those who are financially unable to afford their medications. Moreover, since our model relied on pharmacy data, not medical claims data, patient diagnoses were assumed based on the pharmaceutical treatment regimen. Finally, the model could not be externally validated because we did not have access to patient-level COVID-19 case data, which limited our ability to calculate the sensitivity and specificity of the risk model.

While the interactive map will be useful for multiple purposes, it is for informational purposes only and is not intended to provide medical advice or discourage social distancing or other health-related recommendations. Although Walgreens will take reasonable steps to update this map routinely with the latest available information, SARS-CoV-2 is a novel virus and its spread is rapid and unpredictable. We encourage everyone to visit the CDC’s Coronavirus (COVID-19) webpage for the latest information and recommendations [11]. We encourage the public to contact their health care provider to address any concerns and before taking any personal action in response to the information provided by the model or map.

## Appendix

Multimedia Appendix 1 Specialty medications included in the coronavirus disease (COVID-19) risk calculation.

Multimedia Appendix 2 Maintenance medications included in the coronavirus disease (COVID-19) risk calculation.

## Abbreviations

CDC: Centers for Disease Control and Prevention
COVID-19: coronavirus disease
CSSE: Center for Systems Science and Engineering
ICU: intensive care unit
SARS-CoV-2: severe acute respiratory syndrome coronavirus 2
