# Supplementary material for: Distribution of Patients at Risk for Complications Related to COVID-19 in the United States: Model Development Study
Source: JMIR Public Health Surveill. 2020 Jun 18;6(2):e19606. doi: 10.2196/19606 (PMC7304254; doi:10.2196/19606)
Supplement: Multimedia Appendix 1 [file publichealth_v6i2e19606_app1.docx]

SUPPLEMENTAL FILE

Appendix A. Specialty medications included in the COVID-19 risk calculation

| **Drug Disease Class** | **GPI14** | **GPI Generic Name** |
| --- | --- | --- |
| ASTHMA | 38000030102005 | Ephedrine Sulfate Inj 25 MG/ML |
| ASTHMA | 38000030102010 | Ephedrine Sulfate Inj 50 MG/ML |
| ASTHMA | 42200015001810 | Budesonide Nasal Susp 32 MCG/ACT |
| ASTHMA | 44150010100105 | Cromolyn Sodium Inhal Cap 20 MG |
| ASTHMA | 44150010102505 | Cromolyn Sodium Soln Nebu 20 MG/2ML |
| ASTHMA | 44150010103405 | Cromolyn Sodium Inhal Aerosol Soln 800 MCG/ACT (1 MG/Valve) |
| ASTHMA | 44150050103410 | Nedocromil Sodium Inhal Aerosol 1.75 MG/ACT |
| ASTHMA | 44201010003405 | Albuterol Inhal Aerosol 90 MCG/ACT |
| ASTHMA | 44201010100120 | Albuterol Sulfate Cap For Inhal 200 MCG |
| ASTHMA | 44201010100305 | Albuterol Sulfate Tab 2 MG |
| ASTHMA | 44201010100310 | Albuterol Sulfate Tab 4 MG |
| ASTHMA | 44201010100410 | Albuterol Sulfate Tab CR 4 MG |
| ASTHMA | 44201010100420 | Albuterol Sulfate Tab CR 8 MG |
| ASTHMA | 44201010100470 | Albuterol Sulfate Tab SA OSM 4 MG |
| ASTHMA | 44201010101205 | Albuterol Sulfate Syrup 2 MG/5ML |
| ASTHMA | 44201010102100 | Albuterol Sulfate For Nebu Soln |
| ASTHMA | 44201010102515 | Albuterol Sulfate Soln Nebu 0.083% |
| ASTHMA | 44201010102515 | Albuterol Sulfate Soln Nebu 0.083% (2.5 MG/3ML) |
| ASTHMA | 44201010102520 | Albuterol Sulfate Soln Nebu 0.5% |
| ASTHMA | 44201010102520 | Albuterol Sulfate Soln Nebu 0.5% (5 MG/ML) |
| ASTHMA | 44201010102555 | Albuterol Sulfate Soln Nebu 0.63 MG/3ML (Base Equiv) |
| ASTHMA | 44201010102560 | Albuterol Sulfate Soln Nebu 1.25 MG/3ML (Base Equiv) |
| ASTHMA | 44201010102900 | Albuterol Sulfate Powder |
| ASTHMA | 44201010103410 | Albuterol Sulfate Inhal Aero 108 MCG/ACT (90MCG Base Equiv) |
| ASTHMA | 44201010107410 | Albuterol Sulfate Tab ER 12HR 4 MG |
| ASTHMA | 44201010107410 | Albuterol Sulfate Tab SR 12HR 4 MG |
| ASTHMA | 44201010107420 | Albuterol Sulfate Tab ER 12HR 8 MG |
| ASTHMA | 44201010107420 | Albuterol Sulfate Tab SR 12HR 8 MG |
| ASTHMA | 44201010107470 | Albuterol Sulfate Tab SR 12HR Osmotic 4 MG |
| ASTHMA | 44201010107480 | Albuterol Sulfate Tab SR 12HR Osmotic 8 MG |
| ASTHMA | 44201020102520 | Bitolterol Mesylate Soln Nebu 0.2% |
| ASTHMA | 44201020103405 | Bitolterol Mesylate Inhal Aerosol 0.8% |
| ASTHMA | 44201020103405 | Bitolterol Mesylate Inhal Aerosol 0.8% (0.37 MG/ACT) |
| ASTHMA | 44201027100120 | Formoterol Fumarate Inhal Cap 12 MCG |
| ASTHMA | 44201030102510 | Isoetharine HCl Soln Nebu 0.062% |
| ASTHMA | 44201030102512 | Isoetharine HCl Soln Nebu 0.08% |
| ASTHMA | 44201030102515 | Isoetharine HCl Soln Nebu 0.1% |
| ASTHMA | 44201030102520 | Isoetharine HCl Soln Nebu 0.125% |
| ASTHMA | 44201030102530 | Isoetharine HCl Soln Nebu 0.167% |
| ASTHMA | 44201030102533 | Isoetharine HCl Soln Nebu 0.17% |
| ASTHMA | 44201030102540 | Isoetharine HCl Soln Nebu 0.2% |
| ASTHMA | 44201030102550 | Isoetharine HCl Soln Nebu 0.25% |
| ASTHMA | 44201030102560 | Isoetharine HCl Soln Nebu 1% |
| ASTHMA | 44201030203410 | Isoetharine Mesylate Inhal Aerosol 340 MCG/ACT |
| ASTHMA | 44201040100705 | Isoproterenol HCl SL Tab 10 MG |
| ASTHMA | 44201040102005 | Isoproterenol HCl Inj 0.2 MG/ML |
| ASTHMA | 44201040102520 | Isoproterenol HCl Soln Nebu 0.25% |
| ASTHMA | 44201040102525 | Isoproterenol HCl Soln Nebu 0.5% |
| ASTHMA | 44201040102530 | Isoproterenol HCl Soln Nebu 1% |
| ASTHMA | 44201040103405 | Isoproterenol HCl Inhal Aerosol Soln 131 MCG/ACT |
| ASTHMA | 44201040103405 | Isoproterenol HCl Inhal Aerosol Soln 131 MCG/ACT (2.5 MG/ML) |
| ASTHMA | 44201040103410 | Isoproterenol HCl Inhal Aerosol Soln 0.25% |
| ASTHMA | 44201040203405 | Isoproterenol Sulfate Inhal Aerosol Soln 80 MCG/ACT |
| ASTHMA | 44201045102510 | Levalbuterol HCl Soln Nebu 0.31 MG/3ML (Base Equiv) |
| ASTHMA | 44201045102520 | Levalbuterol HCl Soln Nebu 0.63 MG/3ML (Base Equiv) |
| ASTHMA | 44201045102530 | Levalbuterol HCl Soln Nebu 1.25 MG/3ML (Base Equiv) |
| ASTHMA | 44201045102560 | Levalbuterol HCl Soln Nebu Conc 1.25 MG/0.5ML (Base Equiv) |
| ASTHMA | 44201045503220 | Levalbuterol Tartrate Inhal Aerosol 45 MCG/ACT (Base Equiv) |
| ASTHMA | 44201050200305 | Metaproterenol Sulfate Tab 10 MG |
| ASTHMA | 44201050200310 | Metaproterenol Sulfate Tab 20 MG |
| ASTHMA | 44201050201205 | Metaproterenol Sulfate Syrup 10 MG/5ML |
| ASTHMA | 44201050202503 | Metaproterenol Sulfate Soln Nebu 0.4% |
| ASTHMA | 44201050202505 | Metaproterenol Sulfate Soln Nebu 0.6% |
| ASTHMA | 44201050202510 | Metaproterenol Sulfate Soln Nebu 5% |
| ASTHMA | 44201050203310 | Metaproterenol Sulfate Inhal Aerosol Pow 0.65 MG/ACT |
| ASTHMA | 44201055003220 | Pirbuterol Acetate Inhal Aerosol 200 MCG/ACT |
| ASTHMA | 44201055008120 | Pirbuterol Acetate Breath Activated Inhal Aerosol 200MCG/INH |
| ASTHMA | 44201058103410 | Salmeterol Xinafoate Inhal Aerosol 21 MCG/ACT (25 MCG/Valve) |
| ASTHMA | 44201058108020 | Salmeterol Xinafoate Aer Pow BA 50 MCG/DOSE (Base Equiv) |
| ASTHMA | 44201060200305 | Terbutaline Sulfate Tab 2.5 MG |
| ASTHMA | 44201060200310 | Terbutaline Sulfate Tab 5 MG |
| ASTHMA | 44201060202005 | Terbutaline Sulfate Inj 1 MG/ML |
| ASTHMA | 44201060203405 | Terbutaline Sulfate Inhal Aerosol Soln 0.2 MG/ACT |
| ASTHMA | 44202010050320 | Ephedrine HCl Tab 25 MG |
| ASTHMA | 44202010052900 | Ephedrine HCl Powder |
| ASTHMA | 44202010100110 | Ephedrine Sulfate Cap 25 MG |
| ASTHMA | 44202010100115 | Ephedrine Sulfate Cap 50 MG |
| ASTHMA | 44202010102900 | Ephedrine Sulfate Powder |
| ASTHMA | 44202020001810 | Epinephrine Inj Susp 5 MG/ML |
| ASTHMA | 44202020003410 | Epinephrine Inhal Aerosol 0.22 MG/ACT |
| ASTHMA | 44202020103405 | Epinephrine Bitartrate Inhal Aerosol 0.16 MG/ACT |
| ASTHMA | 44202020103405 | Epinephrine Bitartrate Inhal Aerosol 0.16 MG/ACT(Base Equiv) |
| ASTHMA | 44202020103405 | Epinephrine Bitartrate Inhal Aerosol 0.16MG/ACT (Base Equiv) |
| ASTHMA | 44202020202005 | Epinephrine HCl Inj 0.1 MG/ML |
| ASTHMA | 44202020202010 | Epinephrine HCl Inj 1 MG/ML |
| ASTHMA | 44202020202515 | Epinephrine HCl Soln Nebu 1:100 |
| ASTHMA | 44202020202530 | Epinephrine HCl Soln Nebu 2.25% |
| ASTHMA | 44202020202530 | Epinephrine HCl Soln Nebu 2.25% (1.125% Base Equivalent) |
| ASTHMA | 44202020203410 | Epinephrine HCl Inhal Aerosol 0.27 MG/ACT |
| ASTHMA | 44202030102010 | Ethylnorepinephrine HCl Inj 1 MG/ML |
| ASTHMA | 44202030102020 | Ethylnorepinephrine HCl Inj 2 MG/ML |
| ASTHMA | 44209902103410 | Isoproterenol & Phenylephrine Inhal Aerosol Soln 0.16-0.24 |
| ASTHMA | 44209902413220 | Budesonide-Formoterol Fumarate Dihyd Aerosol 80-4.5 MCG/ACT |
| ASTHMA | 44209902413240 | Budesonide-Formoterol Fumarate Dihyd Aerosol 160-4.5 MCG/ACT |
| ASTHMA | 44209902703250 | Fluticasone-Salmeterol Inhal Aerosol 45-21 MCG/ACT |
| ASTHMA | 44209902703260 | Fluticasone-Salmeterol Inhal Aerosol 115-21 MCG/ACT |
| ASTHMA | 44209902703270 | Fluticasone-Salmeterol Inhal Aerosol 230-21 MCG/ACT |
| ASTHMA | 44300010000305 | Aminophylline Tab 100 MG |
| ASTHMA | 44300010000310 | Aminophylline Tab 200 MG |
| ASTHMA | 44300010000410 | Aminophylline Tab CR 225 MG |
| ASTHMA | 44300010000605 | Aminophylline EC Tab 100 MG |
| ASTHMA | 44300010000610 | Aminophylline EC Tab 200 MG |
| ASTHMA | 44300010002010 | Aminophylline Inj 25 MG/ML |
| ASTHMA | 44300010002060 | Aminophylline Oral Soln 105 MG/5ML |
| ASTHMA | 44300010002900 | Aminophylline Powder |
| ASTHMA | 44300010005205 | Aminophylline Suppos 250 MG |
| ASTHMA | 44300010005210 | Aminophylline Suppos 500 MG |
| ASTHMA | 44300020000310 | Dyphylline Tab 200 MG |
| ASTHMA | 44300020000315 | Dyphylline Tab 400 MG |
| ASTHMA | 44300020001005 | Dyphylline Elixir 100 MG/15ML |
| ASTHMA | 44300020001010 | Dyphylline Elixir 160 MG/15ML |
| ASTHMA | 44300020002005 | Dyphylline Inj 250 MG/ML |
| ASTHMA | 44300030000305 | Oxtriphylline Tab 100 MG |
| ASTHMA | 44300030000310 | Oxtriphylline Tab 200 MG |
| ASTHMA | 44300030000405 | Oxtriphylline Tab CR 400 MG |
| ASTHMA | 44300030000410 | Oxtriphylline Tab CR 600 MG |
| ASTHMA | 44300030001010 | Oxtriphylline Elixir 100 MG/5ML |
| ASTHMA | 44300030001205 | Oxtriphylline Syrup 50 MG/5ML |
| ASTHMA | 44300030007405 | Oxtriphylline Tab SR 12HR 400 MG |
| ASTHMA | 44300030007410 | Oxtriphylline Tab SR 12HR 600 MG |
| ASTHMA | 44300040000105 | Theophylline Cap 100 MG |
| ASTHMA | 44300040000110 | Theophylline Cap 200 MG |
| ASTHMA | 44300040000205 | Theophylline Cap CR 50 MG |
| ASTHMA | 44300040000210 | Theophylline Cap CR 60 MG |
| ASTHMA | 44300040000215 | Theophylline Cap CR 75 MG |
| ASTHMA | 44300040000220 | Theophylline Cap CR 100 MG |
| ASTHMA | 44300040000225 | Theophylline Cap CR 125 MG |
| ASTHMA | 44300040000230 | Theophylline Cap CR 130 MG |
| ASTHMA | 44300040000235 | Theophylline Cap CR 200 MG |
| ASTHMA | 44300040000240 | Theophylline Cap CR 250 MG |
| ASTHMA | 44300040000245 | Theophylline Cap CR 260 MG |
| ASTHMA | 44300040000250 | Theophylline Cap CR 300 MG |
| ASTHMA | 44300040000260 | Theophylline Cap CR 400 MG |
| ASTHMA | 44300040000305 | Theophylline Tab 100 MG |
| ASTHMA | 44300040000310 | Theophylline Tab 125 MG |
| ASTHMA | 44300040000315 | Theophylline Tab 200 MG |
| ASTHMA | 44300040000325 | Theophylline Tab 250 MG |
| ASTHMA | 44300040000330 | Theophylline Tab 300 MG |
| ASTHMA | 44300040000410 | Theophylline Tab CR 100 MG |
| ASTHMA | 44300040000415 | Theophylline Tab CR 200 MG |
| ASTHMA | 44300040000420 | Theophylline Tab CR 250 MG |
| ASTHMA | 44300040000425 | Theophylline Tab CR 300 MG |
| ASTHMA | 44300040000430 | Theophylline Tab CR 400 MG |
| ASTHMA | 44300040000433 | Theophylline Tab CR 450 MG |
| ASTHMA | 44300040000435 | Theophylline Tab CR 500 MG |
| ASTHMA | 44300040001010 | Theophylline Elixir 80 MG/15ML |
| ASTHMA | 44300040001210 | Theophylline Syrup 80 MG/15ML |
| ASTHMA | 44300040002010 | Theophylline Soln 80 MG/15ML |
| ASTHMA | 44300040006910 | Theophylline Cap SR 12HR 50 MG |
| ASTHMA | 44300040006913 | Theophylline Cap SR 12HR 65 MG |
| ASTHMA | 44300040006915 | Theophylline Cap SR 12HR 75 MG |
| ASTHMA | 44300040006920 | Theophylline Cap SR 12HR 100 MG |
| ASTHMA | 44300040006923 | Theophylline Cap SR 12HR 125 MG |
| ASTHMA | 44300040006924 | Theophylline Cap SR 12HR 130 MG |
| ASTHMA | 44300040006930 | Theophylline Cap SR 12HR 200 MG |
| ASTHMA | 44300040006935 | Theophylline Cap SR 12HR 250 MG |
| ASTHMA | 44300040006936 | Theophylline Cap SR 12HR 260 MG |
| ASTHMA | 44300040006940 | Theophylline Cap SR 12HR 300 MG |
| ASTHMA | 44300040007020 | Theophylline Cap ER 24HR 100 MG |
| ASTHMA | 44300040007020 | Theophylline Cap SR 24HR 100 MG |
| ASTHMA | 44300040007030 | Theophylline Cap ER 24HR 200 MG |
| ASTHMA | 44300040007030 | Theophylline Cap SR 24HR 200 MG |
| ASTHMA | 44300040007040 | Theophylline Cap ER 24HR 300 MG |
| ASTHMA | 44300040007040 | Theophylline Cap SR 24HR 300 MG |
| ASTHMA | 44300040007050 | Theophylline Cap ER 24HR 400 MG |
| ASTHMA | 44300040007420 | Theophylline Tab ER 12HR 100 MG |
| ASTHMA | 44300040007420 | Theophylline Tab SR 12HR 100 MG |
| ASTHMA | 44300040007430 | Theophylline Tab CR 200 MG |
| ASTHMA | 44300040007430 | Theophylline Tab ER 12HR 200 MG |
| ASTHMA | 44300040007430 | Theophylline Tab SR 12HR 200 MG |
| ASTHMA | 44300040007435 | Theophylline Tab SR 12HR 250 MG |
| ASTHMA | 44300040007440 | Theophylline Tab CR 300 MG |
| ASTHMA | 44300040007440 | Theophylline Tab ER 12HR 300 MG |
| ASTHMA | 44300040007440 | Theophylline Tab SR 12HR 300 MG |
| ASTHMA | 44300040007455 | Theophylline Tab CR 450 MG |
| ASTHMA | 44300040007455 | Theophylline Tab ER 12HR 450 MG |
| ASTHMA | 44300040007455 | Theophylline Tab SR 12HR 450 MG |
| ASTHMA | 44300040007460 | Theophylline Tab SR 12HR 500 MG |
| ASTHMA | 44300040007540 | Theophylline Tab ER 24HR 400 MG |
| ASTHMA | 44300040007540 | Theophylline Tab SR 24HR 400 MG |
| ASTHMA | 44300040007560 | Theophylline Tab ER 24HR 600 MG |
| ASTHMA | 44300040007560 | Theophylline Tab SR 24HR 600 MG |
| ASTHMA | 44300040012010 | Theophylline in D5W Inj 0.4 MG/ML |
| ASTHMA | 44300040012015 | Theophylline in D5W Inj 0.8 MG/ML |
| ASTHMA | 44300040012020 | Theophylline in D5W Inj 1.6 MG/ML |
| ASTHMA | 44300040012025 | Theophylline in D5W Inj 2 MG/ML |
| ASTHMA | 44300040012027 | Theophylline in D5W Inj 3.2 MG/ML |
| ASTHMA | 44300040012030 | Theophylline in D5W Inj 4 MG/ML |
| ASTHMA | 44400010103408 | Beclomethasone Diprop Inhal Aero Soln 40 MCG/ACT (50/Valve) |
| ASTHMA | 44400010103408 | Beclomethasone Dipropionate Inhal Aero Soln 40 MCG/ACT |
| ASTHMA | 44400010103410 | Beclomethasone Dipropionate Inhal Aero 42 MCG/ACT (50/Valve) |
| ASTHMA | 44400010103428 | Beclomethasone Diprop Inhal Aero Soln 80 MCG/ACT (100/Valve) |
| ASTHMA | 44400010103428 | Beclomethasone Dipropionate Inhal Aero Soln 80 MCG/ACT |
| ASTHMA | 44400010103430 | Beclomethasone Dipropionate Inhal Aero 84 MCG/ACT(100/Valve) |
| ASTHMA | 44400010103430 | Beclomethasone Dipropionate Inhal Aero 84MCG/ACT (100/Valve) |
| ASTHMA | 44400010103450 | Beclomethasone Dipropionate OR/NA Inhaler Aerosol 42 MCG/ACT |
| ASTHMA | 44400015001830 | Budesonide Inhalation Susp 0.25 MG/2ML |
| ASTHMA | 44400015001840 | Budesonide Inhalation Susp 0.5 MG/2ML |
| ASTHMA | 44400015001850 | Budesonide Inhalation Susp 1 MG/2ML |
| ASTHMA | 44400015008009 | Budesonide Inhal Aero Powd 90 MCG/ACT (Breath Activated) |
| ASTHMA | 44400015008018 | Budesonide Inhal Aero Powd 180 MCG/ACT (Breath Activated) |
| ASTHMA | 44400015008020 | Budesonide Inhal Aero Powd 200 MCG/INH (Breath Activated) |
| ASTHMA | 44400017003420 | Ciclesonide Inhal Aerosol 80 MCG/ACT |
| ASTHMA | 44400017003440 | Ciclesonide Inhal Aerosol 160 MCG/ACT |
| ASTHMA | 44400020102410 | Dexamethasone Sodium Phosphate Inh 84 MCG/DOSE |
| ASTHMA | 44400030003400 | Flunisolide Inhal Aerosol 250 MCG/ACT |
| ASTHMA | 44400030123420 | Flunisolide HFA Inhal Aerosol 80 MCG/ACT |
| ASTHMA | 44400033203220 | Fluticasone Propionate Inhal Aerosol 44 MCG/ACT (50/Valve) |
| ASTHMA | 44400033203230 | Fluticasone Propionate Inhal Aerosol 110 MCG/ACT (125/Valve) |
| ASTHMA | 44400033203240 | Fluticasone Propionate Inhal Aerosol 220 MCG/ACT (250/Valve) |
| ASTHMA | 44400033208010 | Fluticasone Propionate Aer Pow BA 50 MCG/BLISTER |
| ASTHMA | 44400033208020 | Fluticasone Propionate Aer Pow BA 100 MCG/BLISTER |
| ASTHMA | 44400033208030 | Fluticasone Propionate Aer Pow BA 250 MCG/BLISTER |
| ASTHMA | 44400033223220 | Fluticasone Propionate HFA Inhal Aero 44 MCG/ACT (50/Valve) |
| ASTHMA | 44400033223220 | Fluticasone Propionate HFA Inhal Aerosol 44 MCG/ACT |
| ASTHMA | 44400033223230 | Fluticasone Propionate HFA Inhal Aer 110 MCG/ACT (125/Valve) |
| ASTHMA | 44400033223230 | Fluticasone Propionate HFA Inhal Aerosol 110 MCG/ACT |
| ASTHMA | 44400033223240 | Fluticasone Propionate HFA Inhal Aer 220 MCG/ACT (250/Valve) |
| ASTHMA | 44400033223240 | Fluticasone Propionate HFA Inhal Aerosol 220 MCG/ACT |
| ASTHMA | 44400036208010 | Mometasone Furoate Inhal Powd 110 MCG/INH (Breath Activated) |
| ASTHMA | 44400036208020 | Mometasone Furoate Inhal Powd 220 MCG/INH (Breath Activated) |
| ASTHMA | 44400040203405 | Triamcinolone Acetonide Inhal Aerosol 100 MCG/ACT |
| ASTHMA | 44400040203405 | Triamcinolone Acetonide Inhal Aerosol 100 MCG/ACT(200/Valve) |
| ASTHMA | 44400040203405 | Triamcinolone Acetonide Inhal Aerosol 100MCG/ACT (200/Valve) |
| ASTHMA | 44400040203405 | Triamcinolone Acetonide Inhal Aerosol 75 MCG/ACT (200/Valve) |
| ASTHMA | 44504085000330 | Zileuton Tab 600 MG |
| ASTHMA | 44504085007420 | Zileuton Tab ER 12HR 600 MG |
| ASTHMA | 44504085007420 | Zileuton Tab SR 12HR 600 MG |
| ASTHMA | 44505050100330 | Montelukast Sodium Tab 10 MG (Base Equiv) |
| ASTHMA | 44505050100516 | Montelukast Sodium Chew Tab 4 MG (Base Equiv) |
| ASTHMA | 44505050100520 | Montelukast Sodium Chew Tab 5 MG (Base Equiv) |
| ASTHMA | 44505050103020 | Montelukast Sodium Oral Granules Packet 4 MG (Base Equiv) |
| ASTHMA | 44505080000310 | Zafirlukast Tab 10 MG |
| ASTHMA | 44505080000320 | Zafirlukast Tab 20 MG |
| ASTHMA | 44603060002120 | Omalizumab For Inj 150 MG |
| ASTHMA | 44991002100310 | Aminophylline-GG Tab 130-100 MG |
| ASTHMA | 44991002120310 | Aminophylline-KI Tab 130-195 MG |
| ASTHMA | 44991002200110 | Dyphylline-GG Cap 200-100 MG |
| ASTHMA | 44991002200110 | Dyphylline-Guaifenesin Cap 200-100 MG |
| ASTHMA | 44991002200115 | Dyphylline-GG Cap 200-200 MG |
| ASTHMA | 44991002200310 | Dyphylline-GG Tab 200-100 MG |
| ASTHMA | 44991002200315 | Dyphylline-GG Tab 200-200 MG |
| ASTHMA | 44991002200315 | Dyphylline-Guaifenesin Tab 200-200 MG |
| ASTHMA | 44991002200320 | Dyphylline-Guaifenesin Tab 200-300 MG |
| ASTHMA | 44991002200325 | Dyphylline-Guaifenesin Tab 200-400 MG |
| ASTHMA | 44991002200910 | Dyphylline-GG Liq 100-100 MG/15ML |
| ASTHMA | 44991002200910 | Dyphylline-Guaifenesin Liqd 100-100 MG/15ML |
| ASTHMA | 44991002200915 | Dyphylline-GG Liq 200-200 MG/15ML |
| ASTHMA | 44991002200920 | Dyphylline-GG Liq 300-300 MG/15ML |
| ASTHMA | 44991002200920 | Dyphylline-Guaifenesin Liqd 100-100 MG/5ML |
| ASTHMA | 44991002201010 | Dyphylline-GG Elixir 100-100 MG/15ML |
| ASTHMA | 44991002201010 | Dyphylline-Guaifenesin Elixir 100-100 MG/15ML |
| ASTHMA | 44991002201215 | Dyphylline-Guaifenesin Syrup 200-100 MG/5ML |
| ASTHMA | 44991002300315 | Oxtriphylline-GG Tab 200-100 MG |
| ASTHMA | 44991002301010 | Oxtriphylline-GG Elixir 300-150 MG/15ML |
| ASTHMA | 44991002301010 | Oxtriphylline-Guaifenesin Elixir 300-150 MG/15ML |
| ASTHMA | 44991002400110 | Theophylline-GG Cap 150-90 MG |
| ASTHMA | 44991002400110 | Theophylline-Guaifenesin Cap 150-90 MG |
| ASTHMA | 44991002400130 | Theophylline-GG Cap 300-180 MG |
| ASTHMA | 44991002400130 | Theophylline-Guaifenesin Cap 300-180 MG |
| ASTHMA | 44991002400323 | Theophylline-Guaifenesin Tab 150-200 MG |
| ASTHMA | 44991002400925 | Theophylline-Guaifenesin Liquid 150-90 MG/15ML |
| ASTHMA | 44991002401025 | Theophylline-GG Elixir 150-90 MG/15ML |
| ASTHMA | 44991002401025 | Theophylline-Guaifenesin Elixir 150-90 MG/15ML |
| ASTHMA | 44991002401225 | Theophylline-GG Syrup 150-90 MG/15ML |
| ASTHMA | 44991002402015 | Theophylline-Guaifenesin Soln 100-100 MG/15ML |
| ASTHMA | 44991002402025 | Theophylline-GG Soln 150-90 MG/15ML |
| ASTHMA | 44991002402030 | Theophylline-Guaifenesin Soln 150-200 MG/15ML |
| ASTHMA | 44991002421010 | Theophylline-KI Elixir 130-80 MG/15ML |
| ASTHMA | 44991002421010 | Theophylline-KI Elixir 80-130 MG/15ML |
| ASTHMA | 44991002442010 | Theophylline-Iodinated Glycerol Soln 120-30 MG/15ML |
| ASTHMA | 44991002500310 | Theophylline Sod Glycinate-GG Tab 300-100 MG |
| ASTHMA | 44991002501010 | Theophylline Sod Glycinate-GG Elixir 300-100 MG/15ML |
| ASTHMA | 44991002501210 | Theophylline Sod Glycinate-GG Syrup 300-100 MG/15ML |
| ASTHMA | 44992002100310 | Theophylline-Ephedrine Tab 130-24 MG |
| ASTHMA | 44992203100305 | Theophylline-Ephedrine-GG Tab 60-12.5-100 MG |
| ASTHMA | 44992203150310 | Theophylline-PSE-GG Tab 150-30-150 MG |
| ASTHMA | 44992203151010 | Theophylline-PSE-GG Elixir 150-30-150 MG/15ML |
| ASTHMA | 44992204101010 | Isoproterenol Compound Elixir |
| ASTHMA | 44993003100110 | Aminophylline-Ephedrine-Amobarbital Cap 130-25-25 MG |
| ASTHMA | 44993003220305 | Theophylline-Ephedrine-Phenobarbital Tab 118-24-8 MG |
| ASTHMA | 44993003220310 | Theophylline-Ephedrine-Phenobarbital Tab 130-24-8 MG |
| ASTHMA | 44993003220420 | Theophylline-Ephedrine-Phenobarbital Tab CR 180-48-25 MG |
| ASTHMA | 44993003221820 | Theophylline-Ephedrine-Phenobarbital Susp 65-12-4 MG/5ML |
| ASTHMA | 44993003300310 | Theophylline-Ephedrine-Hydroxyzine Tab 97.5-18.75-7.5 MG |
| ASTHMA | 44993003300320 | Theophylline-Ephedrine-Hydroxyzine Tab 130-25-10 MG |
| ASTHMA | 44993003301210 | Theophylline-Ephedrine-Hydroxyzine Syrup 97-18-7.5 MG/15ML |
| ASTHMA | 44993204100310 | Aminophylline-Ephedrine-PB-GG Tab 130-16-8-100 MG |
| ASTHMA | 44993204120310 | Aminophylline-Ephedrine-PB-KI Tab 130-16-8-195 MG |
| ASTHMA | 44993204200310 | Dyphylline-Ephedrine-PB-GG Tab 100-16-16-200 MG |
| ASTHMA | 44993204201010 | Dyphylline-Ephedrine-PB-GG Elixir 150-24-24-300 MG/15ML |
| ASTHMA | 44993204300310 | Theo Cal Sal-Ephedrine-PB-KI Tab 130-24-24-320 MG |
| ASTHMA | 44993204350110 | Theophylline-Ephed-Butabarb-GG Cap 150-25-20-100 MG |
| ASTHMA | 44993204351010 | Theophylline-Ephed-Butabarb-GG Elixir 150-25-20-100 MG/15ML |
| ASTHMA | 44993204400320 | Theophylline-Ephedrine-PB-GG Tab 100-24-8-100 MG |
| ASTHMA | 44993204401010 | Theophylline-Ephedrine-PB-GG Elixir 30-24-8-100 MG/10ML |
| ASTHMA | 44993204401015 | Theophylline-Ephedrine-PB-GG Elixir 45-12-7.5-78 MG/10ML |
| ASTHMA | 44993204401020 | Theophylline-Ephedrine-PB-GG Elixir 60-12-7.5-78 MG/15ML |
| ASTHMA | 44993204501020 | Theophylline-PSE-Butabarb-GG Elixir 150-30-15-150 MG/5ML |
| ASTHMA | 44994002121210 | Ephedrine-KI Syrup 8-150 MG/5ML |
| ASTHMA | 44994002201210 | Isoproterenol w/ Cal Iodide Syrup 3-150 MG/5ML |
| ASTHMA | 44999004500320 | Theophylline-Ephedrine-Pyrilamine-GG Tab 120-24-16-100 MG |
| ASTHMA UNSPECIFIED | 44400036208010 | Mometasone Furoate Inhal Powd 110 MCG/INH (Breath Activated) |
| ASTHMA UNSPECIFIED | 44400036208020 | Mometasone Furoate Inhal Powd 220 MCG/INH (Breath Activated) |
| ASTHMA UNSPECIFIED | 44991002402030 | Theophylline-Guaifenesin Soln 150-200 MG/15ML |
| Alpha-1 Proteinase Inhibitors | 45100010102015 | Alpha1-Proteinase Inhibitor (Human) |
| Alpha-1 Proteinase Inhibitors | 45100010102020 | Alpha1-Proteinase Inhibitor (Human) |
| Alpha-1 Proteinase Inhibitors | 45100010102110 | Alpha1-Proteinase Inhibitor (Human) |
| Alpha-1 Proteinase Inhibitors | 45100010102118 | Alpha1-Proteinase Inhibitor (Human) |
| Alpha-1 Proteinase Inhibitors | 45100010102120 | Alpha1-Proteinase Inhibitor (Human) |
| Anti-Infectives - misc | 3530025000320 | Fidaxomicin |
| Anti-Infectives - misc | 4200050200320 | Omadacycline Tosylate |
| Anti-Infectives - misc | 4200050202120 | Omadacycline Tosylate |
| Anti-Infectives - misc | 12200030102030 | Ganciclovir Sodium |
| Anti-Infectives - misc | 12200030102110 | Ganciclovir Sodium |
| Anti-Infectives - misc | 12200066100320 | Valganciclovir HCl |
| Anti-Infectives - misc | 12200066102120 | Valganciclovir HCl |
| Anti-Infectives - misc | 12604075002120 | Ribavirin |
| Anti-Infectives - misc | 19503015002020 | Bezlotoxumab |
| Anticoagulant | 83101010102015 | Dalteparin Sodium |
| Anticoagulant | 83101010102020 | Dalteparin Sodium |
| Anticoagulant | 83101010102040 | Dalteparin Sodium |
| Anticoagulant | 83101010102045 | Dalteparin Sodium |
| Anticoagulant | 83101010102053 | Dalteparin Sodium |
| Anticoagulant | 83101010102056 | Dalteparin Sodium |
| Anticoagulant | 83101010102060 | Dalteparin Sodium |
| Anticoagulant | 83101010102080 | Dalteparin Sodium |
| Anticoagulant | 83101020102012 | Enoxaparin Sodium |
| Anticoagulant | 83101020102013 | Enoxaparin Sodium |
| Anticoagulant | 83101020102014 | Enoxaparin Sodium |
| Anticoagulant | 83101020102015 | Enoxaparin Sodium |
| Anticoagulant | 83101020102016 | Enoxaparin Sodium |
| Anticoagulant | 83101020102018 | Enoxaparin Sodium |
| Anticoagulant | 83101020102020 | Enoxaparin Sodium |
| Anticoagulant | 83101020102050 | Enoxaparin Sodium |
| Anticoagulant | 83103030102020 | Fondaparinux Sodium |
| Anticoagulant | 83103030102035 | Fondaparinux Sodium |
| Anticoagulant | 83103030102040 | Fondaparinux Sodium |
| Anticoagulant | 83103030102045 | Fondaparinux Sodium |
| Asthma | 44603060002120 | Omalizumab |
| Asthma | 4460306000E510 | Omalizumab |
| Asthma | 4460306000E520 | Omalizumab |
| Asthma | 4460402000E520 | Benralizumab |
| Asthma | 44604055002120 | Mepolizumab |
| Asthma | 4460405500D530 | Mepolizumab |
| Asthma | 4460405500E530 | Mepolizumab |
| Asthma | 44604460002020 | Reslizumab |
| Asthma | 9027302000E515 | Dupilumab |
| Bleeding Disorder - misc | 77204030000305 | Phytonadione |
| Bleeding Disorder - misc | 82405010200320 | Avatrombopag Maleate |
| Bleeding Disorder - misc | 82405030100310 | Eltrombopag Olamine |
| Bleeding Disorder - misc | 82405030100320 | Eltrombopag Olamine |
| Bleeding Disorder - misc | 82405030100330 | Eltrombopag Olamine |
| Bleeding Disorder - misc | 82405030100340 | Eltrombopag Olamine |
| Bleeding Disorder - misc | 82405030103030 | Eltrombopag Olamine |
| Bleeding Disorder - misc | 82405045000320 | Lusutrombopag |
| Bleeding Disorder - misc | 82405060002110 | Romiplostim |
| Bleeding Disorder - misc | 82405060002120 | Romiplostim |
| Bleeding Disorder - misc | 82405060002130 | Romiplostim |
| Bleeding Disorder - misc | 84100040000320 | Tranexamic Acid |
| Bleeding Disorder - misc | 85100031002120 | Coagulation Factor X (Human) |
| Bleeding Disorder - misc | 85100031002140 | Coagulation Factor X (Human) |
| Bleeding Disorder - misc | 85100035002120 | Fibrinogen Concentrate (Human) |
| Blood Modifier | 82300062002030 | Ferric Carboxymaltose |
| Blood Modifier | 82400540102120 | Luspatercept-aamt |
| Blood Modifier | 82400540102140 | Luspatercept-aamt |
| Blood Modifier | 82401015102010 | Darbepoetin Alfa |
| Blood Modifier | 82401015102020 | Darbepoetin Alfa |
| Blood Modifier | 82401015102030 | Darbepoetin Alfa |
| Blood Modifier | 82401015102040 | Darbepoetin Alfa |
| Blood Modifier | 82401015102060 | Darbepoetin Alfa |
| Blood Modifier | 82401015102070 | Darbepoetin Alfa |
| Blood Modifier | 8240101510E510 | Darbepoetin Alfa |
| Blood Modifier | 8240101510E528 | Darbepoetin Alfa |
| Blood Modifier | 8240101510E543 | Darbepoetin Alfa |
| Blood Modifier | 8240101510E552 | Darbepoetin Alfa |
| Blood Modifier | 8240101510E560 | Darbepoetin Alfa |
| Blood Modifier | 8240101510E575 | Darbepoetin Alfa |
| Blood Modifier | 8240101510E582 | Darbepoetin Alfa |
| Blood Modifier | 8240101510E588 | Darbepoetin Alfa |
| Blood Modifier | 8240101510E590 | Darbepoetin Alfa |
| Blood Modifier | 82401020002010 | Epoetin Alfa |
| Blood Modifier | 82401020002015 | Epoetin Alfa |
| Blood Modifier | 82401020002020 | Epoetin Alfa |
| Blood Modifier | 82401020002040 | Epoetin Alfa |
| Blood Modifier | 82401020002050 | Epoetin Alfa |
| Blood Modifier | 82401020002060 | Epoetin Alfa |
| Blood Modifier | 82401020042010 | Epoetin Alfa-epbx |
| Blood Modifier | 82401020042015 | Epoetin Alfa-epbx |
| Blood Modifier | 82401020042020 | Epoetin Alfa-epbx |
| Blood Modifier | 82401020042040 | Epoetin Alfa-epbx |
| Blood Modifier | 82401020042060 | Epoetin Alfa-epbx |
| Blood Modifier | 8240104010E510 | Methoxy Polyethylene Glycol-Epoetin Beta |
| Blood Modifier | 8240104010E515 | Methoxy Polyethylene Glycol-Epoetin Beta |
| Blood Modifier | 8240104010E520 | Methoxy Polyethylene Glycol-Epoetin Beta |
| Blood Modifier | 8240104010E525 | Methoxy Polyethylene Glycol-Epoetin Beta |
| Blood Modifier | 8240104010E535 | Methoxy Polyethylene Glycol-Epoetin Beta |
| Blood Modifier | 8240104010E545 | Methoxy Polyethylene Glycol-Epoetin Beta |
| Blood Modifier | 82401520002010 | Filgrastim |
| Blood Modifier | 82401520002012 | Filgrastim |
| Blood Modifier | 8240152000E545 | Filgrastim |
| Blood Modifier | 8240152000E550 | Filgrastim |
| Blood Modifier | 82401520102020 | Filgrastim-aafi |
| Blood Modifier | 82401520102030 | Filgrastim-aafi |
| Blood Modifier | 8240152010E520 | Filgrastim-aafi |
| Blood Modifier | 8240152010E530 | Filgrastim-aafi |
| Blood Modifier | 8240152060E530 | Filgrastim-sndz |
| Blood Modifier | 8240152060E540 | Filgrastim-sndz |
| Blood Modifier | 82401520702020 | Tbo-Filgrastim |
| Blood Modifier | 82401520702030 | Tbo-Filgrastim |
| Blood Modifier | 8240152070E530 | Tbo-Filgrastim |
| Blood Modifier | 8240152070E540 | Tbo-Filgrastim |
| Blood Modifier | 8240157000E520 | Pegfilgrastim |
| Blood Modifier | 8240157000F820 | Pegfilgrastim |
| Blood Modifier | 8240157005E520 | Pegfilgrastim-bmez |
| Blood Modifier | 8240157010E520 | Pegfilgrastim-cbqv |
| Blood Modifier | 8240157020E520 | Pegfilgrastim-jmdb |
| Blood Modifier | 82402050002120 | Sargramostim |
| Blood Modifier | 85756040100310 | Fostamatinib Disodium |
| Blood Modifier | 85756040100320 | Fostamatinib Disodium |
| Chronic Inflammatory Disease | 52503080002120 | Vedolizumab |
| Chronic Inflammatory Disease | 52504070002020 | Ustekinumab (IV) |
| Chronic Inflammatory Disease | 52505020106420 | Certolizumab Pegol |
| Chronic Inflammatory Disease | 52505020106440 | Certolizumab Pegol |
| Chronic Inflammatory Disease | 52505020106460 | Certolizumab Pegol |
| Chronic Inflammatory Disease | 52505040002120 | Infliximab |
| Chronic Inflammatory Disease | 52505040102120 | Infliximab-abda |
| Chronic Inflammatory Disease | 52505040202120 | Infliximab-dyyb |
| Chronic Inflammatory Disease | 6625005000D510 | Methotrexate (Antirheumatic) |
| Chronic Inflammatory Disease | 6625005000D511 | Methotrexate (Antirheumatic) |
| Chronic Inflammatory Disease | 6625005000D512 | Methotrexate (Antirheumatic) |
| Chronic Inflammatory Disease | 6625005000D515 | Methotrexate (Antirheumatic) |
| Chronic Inflammatory Disease | 6625005000D517 | Methotrexate (Antirheumatic) |
| Chronic Inflammatory Disease | 6625005000D518 | Methotrexate (Antirheumatic) |
| Chronic Inflammatory Disease | 6625005000D519 | Methotrexate (Antirheumatic) |
| Chronic Inflammatory Disease | 6625005000D520 | Methotrexate (Antirheumatic) |
| Chronic Inflammatory Disease | 6625005000D522 | Methotrexate (Antirheumatic) |
| Chronic Inflammatory Disease | 6625005000D523 | Methotrexate (Antirheumatic) |
| Chronic Inflammatory Disease | 6625005000D525 | Methotrexate (Antirheumatic) |
| Chronic Inflammatory Disease | 6625005000D527 | Methotrexate (Antirheumatic) |
| Chronic Inflammatory Disease | 6625005000D528 | Methotrexate (Antirheumatic) |
| Chronic Inflammatory Disease | 6625005000D530 | Methotrexate (Antirheumatic) |
| Chronic Inflammatory Disease | 6625005000D535 | Methotrexate (Antirheumatic) |
| Chronic Inflammatory Disease | 6625005000D540 | Methotrexate (Antirheumatic) |
| Chronic Inflammatory Disease | 6625005000D545 | Methotrexate (Antirheumatic) |
| Chronic Inflammatory Disease | 6626001000E520 | Anakinra |
| Chronic Inflammatory Disease | 6627001500F420 | Adalimumab |
| Chronic Inflammatory Disease | 6627001500F430 | Adalimumab |
| Chronic Inflammatory Disease | 6627001500F440 | Adalimumab |
| Chronic Inflammatory Disease | 6627001500F450 | Adalimumab |
| Chronic Inflammatory Disease | 6627001500F804 | Adalimumab |
| Chronic Inflammatory Disease | 6627001500F805 | Adalimumab |
| Chronic Inflammatory Disease | 6627001500F809 | Adalimumab |
| Chronic Inflammatory Disease | 6627001500F810 | Adalimumab |
| Chronic Inflammatory Disease | 6627001500F820 | Adalimumab |
| Chronic Inflammatory Disease | 6627001500F830 | Adalimumab |
| Chronic Inflammatory Disease | 6627001500F840 | Adalimumab |
| Chronic Inflammatory Disease | 6627001500F880 | Adalimumab |
| Chronic Inflammatory Disease | 66270040002015 | Golimumab |
| Chronic Inflammatory Disease | 6627004000D520 | Golimumab |
| Chronic Inflammatory Disease | 6627004000D540 | Golimumab |
| Chronic Inflammatory Disease | 6627004000E520 | Golimumab |
| Chronic Inflammatory Disease | 6627004000E540 | Golimumab |
| Chronic Inflammatory Disease | 66290030002120 | Etanercept |
| Chronic Inflammatory Disease | 6629003000D530 | Etanercept |
| Chronic Inflammatory Disease | 6629003000E525 | Etanercept |
| Chronic Inflammatory Disease | 6629003000E530 | Etanercept |
| Chronic Inflammatory Disease | 66400010002120 | Abatacept |
| Chronic Inflammatory Disease | 6640001000D520 | Abatacept |
| Chronic Inflammatory Disease | 6640001000E510 | Abatacept |
| Chronic Inflammatory Disease | 6640001000E515 | Abatacept |
| Chronic Inflammatory Disease | 6640001000E520 | Abatacept |
| Chronic Inflammatory Disease | 6650006000D520 | Sarilumab |
| Chronic Inflammatory Disease | 6650006000D530 | Sarilumab |
| Chronic Inflammatory Disease | 6650006000E520 | Sarilumab |
| Chronic Inflammatory Disease | 6650006000E530 | Sarilumab |
| Chronic Inflammatory Disease | 66500070002030 | Tocilizumab |
| Chronic Inflammatory Disease | 66500070002035 | Tocilizumab |
| Chronic Inflammatory Disease | 66500070002040 | Tocilizumab |
| Chronic Inflammatory Disease | 6650007000D520 | Tocilizumab |
| Chronic Inflammatory Disease | 6650007000E520 | Tocilizumab |
| Chronic Inflammatory Disease | 66603010000310 | Baricitinib |
| Chronic Inflammatory Disease | 66603010000320 | Baricitinib |
| Chronic Inflammatory Disease | 66603065100320 | Tofacitinib Citrate |
| Chronic Inflammatory Disease | 66603065100330 | Tofacitinib Citrate |
| Chronic Inflammatory Disease | 66603065107530 | Tofacitinib Citrate |
| Chronic Inflammatory Disease | 66603065107550 | Tofacitinib Citrate |
| Chronic Inflammatory Disease | 66603072007520 | Upadacitinib |
| Chronic Inflammatory Disease | 66700015000330 | Apremilast |
| Chronic Inflammatory Disease | 6670001500B720 | Apremilast |
| Chronic Inflammatory Disease | 9025052000E520 | Brodalumab |
| Chronic Inflammatory Disease | 9025054200D220 | Guselkumab |
| Chronic Inflammatory Disease | 9025054200E520 | Guselkumab |
| Chronic Inflammatory Disease | 9025055400D520 | Ixekizumab |
| Chronic Inflammatory Disease | 9025055400E520 | Ixekizumab |
| Chronic Inflammatory Disease | 9025057070F820 | Risankizumab-rzaa |
| Chronic Inflammatory Disease | 9025057500D520 | Secukinumab |
| Chronic Inflammatory Disease | 9025057500D530 | Secukinumab |
| Chronic Inflammatory Disease | 9025057500E520 | Secukinumab |
| Chronic Inflammatory Disease | 9025057500E530 | Secukinumab |
| Chronic Inflammatory Disease | 9025058010E520 | Tildrakizumab-asmn |
| Chronic Inflammatory Disease | 90250585002020 | Ustekinumab |
| Chronic Inflammatory Disease | 9025058500E520 | Ustekinumab |
| Chronic Inflammatory Disease | 9025058500E540 | Ustekinumab |
| Chronic Inflammatory Disease | 9027302000E520 | Dupilumab |
| Chronic Inflammatory Disease | 99422015002120 | Belimumab |
| Chronic Inflammatory Disease | 99422015002140 | Belimumab |
| Chronic Inflammatory Disease | 9942201500D520 | Belimumab |
| Chronic Inflammatory Disease | 9942201500E520 | Belimumab |
| Cystic Fibrosis | 7000010121830 | Amikacin Sulfate Liposome |
| Cystic Fibrosis | 7000070000120 | Tobramycin |
| Cystic Fibrosis | 7000070002520 | Tobramycin |
| Cystic Fibrosis | 7000070002530 | Tobramycin |
| Cystic Fibrosis | 16100004202132 | Colistimethate Sodium |
| Cystic Fibrosis | 16140010402120 | Aztreonam Lysine |
| Cystic Fibrosis | 45302030000320 | Ivacaftor |
| Cystic Fibrosis | 45302030003010 | Ivacaftor |
| Cystic Fibrosis | 45302030003020 | Ivacaftor |
| Cystic Fibrosis | 45302030003030 | Ivacaftor |
| Cystic Fibrosis | 45304020002010 | Dornase Alfa |
| Cystic Fibrosis | 45309902300310 | Lumacaftor-Ivacaftor |
| Cystic Fibrosis | 45309902300320 | Lumacaftor-Ivacaftor |
| Cystic Fibrosis | 45309902303010 | Lumacaftor-Ivacaftor |
| Cystic Fibrosis | 45309902303020 | Lumacaftor-Ivacaftor |
| Cystic Fibrosis | 4530990280B710 | Tezacaftor-Ivacaftor |
| Cystic Fibrosis | 4530990280B720 | Tezacaftor-Ivacaftor |
| Cystic Fibrosis | 4530990340B740 | Elexacaftor-Tezacaftor-Ivacaftor |
| Cystic Fibrosis | 51200024000330 | Pancrelipase (Lipase-Protease-Amylase) |
| Cystic Fibrosis | 51200024000360 | Pancrelipase (Lipase-Protease-Amylase) |
| Cystic Fibrosis | 51200024006704 | Pancrelipase (Lipase-Protease-Amylase) |
| Cystic Fibrosis | 51200024006705 | Pancrelipase (Lipase-Protease-Amylase) |
| Cystic Fibrosis | 51200024006707 | Pancrelipase (Lipase-Protease-Amylase) |
| Cystic Fibrosis | 51200024006709 | Pancrelipase (Lipase-Protease-Amylase) |
| Cystic Fibrosis | 51200024006710 | Pancrelipase (Lipase-Protease-Amylase) |
| Cystic Fibrosis | 51200024006714 | Pancrelipase (Lipase-Protease-Amylase) |
| Cystic Fibrosis | 51200024006720 | Pancrelipase (Lipase-Protease-Amylase) |
| Cystic Fibrosis | 51200024006725 | Pancrelipase (Lipase-Protease-Amylase) |
| Cystic Fibrosis | 51200024006728 | Pancrelipase (Lipase-Protease-Amylase) |
| Cystic Fibrosis | 51200024006734 | Pancrelipase (Lipase-Protease-Amylase) |
| Cystic Fibrosis | 51200024006740 | Pancrelipase (Lipase-Protease-Amylase) |
| Cystic Fibrosis | 51200024006747 | Pancrelipase (Lipase-Protease-Amylase) |
| Cystic Fibrosis | 51200024006749 | Pancrelipase (Lipase-Protease-Amylase) |
| Cystic Fibrosis | 51200024006750 | Pancrelipase (Lipase-Protease-Amylase) |
| Cystic Fibrosis | 51200024006751 | Pancrelipase (Lipase-Protease-Amylase) |
| Cystic Fibrosis | 51200024006754 | Pancrelipase (Lipase-Protease-Amylase) |
| Cystic Fibrosis | 51200024006760 | Pancrelipase (Lipase-Protease-Amylase) |
| Cystic Fibrosis | 51200024006762 | Pancrelipase (Lipase-Protease-Amylase) |
| Cystic Fibrosis | 51200024006772 | Pancrelipase (Lipase-Protease-Amylase) |
| Cystic Fibrosis | 51200024006780 | Pancrelipase (Lipase-Protease-Amylase) |
| Cystic Fibrosis | 51200024006783 | Pancrelipase (Lipase-Protease-Amylase) |
| HIV Supportive | 13000040000310 | Pyrimethamine |
| HIV Supportive | 21404020100305 | Megestrol Acetate |
| HIV Supportive | 21404020100310 | Megestrol Acetate |
| HIV Supportive | 21404020101810 | Megestrol Acetate |
| HIV Supportive | 26000023201840 | Megestrol Acetate (Appetite) |
| HIV Supportive | 30100020102118 | Somatropin (Non-Refrigerated) |
| HIV Supportive | 30100020102121 | Somatropin (Non-Refrigerated) |
| HIV Supportive | 30100020102125 | Somatropin (Non-Refrigerated) |
| HIV Supportive | 30150085102120 | Tesamorelin Acetate |
| HIV Supportive | 30150085102130 | Tesamorelin Acetate |
| HIV Supportive | 47250025000620 | Crofelemer |
| HIV/AIDS | 12102060000305 | Maraviroc |
| HIV/AIDS | 12102060000310 | Maraviroc |
| HIV/AIDS | 12102060000320 | Maraviroc |
| HIV/AIDS | 12102060000330 | Maraviroc |
| HIV/AIDS | 12102060002020 | Maraviroc |
| HIV/AIDS | 12102240302020 | Ibalizumab-uiyk |
| HIV/AIDS | 12102530002120 | Enfuvirtide |
| HIV/AIDS | 12103015100305 | Dolutegravir Sodium |
| HIV/AIDS | 12103015100310 | Dolutegravir Sodium |
| HIV/AIDS | 12103015100320 | Dolutegravir Sodium |
| HIV/AIDS | 12103020000310 | Elvitegravir |
| HIV/AIDS | 12103020000320 | Elvitegravir |
| HIV/AIDS | 12103060100320 | Raltegravir Potassium |
| HIV/AIDS | 12103060100330 | Raltegravir Potassium |
| HIV/AIDS | 12103060100510 | Raltegravir Potassium |
| HIV/AIDS | 12103060100540 | Raltegravir Potassium |
| HIV/AIDS | 12103060103020 | Raltegravir Potassium |
| HIV/AIDS | 12104515200130 | Atazanavir Sulfate |
| HIV/AIDS | 12104515200140 | Atazanavir Sulfate |
| HIV/AIDS | 12104515200150 | Atazanavir Sulfate |
| HIV/AIDS | 12104515203020 | Atazanavir Sulfate |
| HIV/AIDS | 12104520100310 | Darunavir Ethanolate |
| HIV/AIDS | 12104520100315 | Darunavir Ethanolate |
| HIV/AIDS | 12104520100340 | Darunavir Ethanolate |
| HIV/AIDS | 12104520100350 | Darunavir Ethanolate |
| HIV/AIDS | 12104520101820 | Darunavir Ethanolate |
| HIV/AIDS | 12104525100330 | Fosamprenavir Calcium |
| HIV/AIDS | 12104525101820 | Fosamprenavir Calcium |
| HIV/AIDS | 12104530200120 | Indinavir Sulfate |
| HIV/AIDS | 12104530200140 | Indinavir Sulfate |
| HIV/AIDS | 12104545200320 | Nelfinavir Mesylate |
| HIV/AIDS | 12104545200340 | Nelfinavir Mesylate |
| HIV/AIDS | 12104560000120 | Ritonavir |
| HIV/AIDS | 12104560000320 | Ritonavir |
| HIV/AIDS | 12104560002020 | Ritonavir |
| HIV/AIDS | 12104560003020 | Ritonavir |
| HIV/AIDS | 12104580200120 | Saquinavir Mesylate |
| HIV/AIDS | 12104580200320 | Saquinavir Mesylate |
| HIV/AIDS | 12104585000120 | Tipranavir |
| HIV/AIDS | 12104585002020 | Tipranavir |
| HIV/AIDS | 12105005100320 | Abacavir Sulfate |
| HIV/AIDS | 12105005102020 | Abacavir Sulfate |
| HIV/AIDS | 12105015002120 | Didanosine |
| HIV/AIDS | 12105015002140 | Didanosine |
| HIV/AIDS | 12105015006520 | Didanosine |
| HIV/AIDS | 12105015006528 | Didanosine |
| HIV/AIDS | 12105015006535 | Didanosine |
| HIV/AIDS | 12105015006550 | Didanosine |
| HIV/AIDS | 12106030000120 | Emtricitabine |
| HIV/AIDS | 12106030002010 | Emtricitabine |
| HIV/AIDS | 12106060000320 | Lamivudine |
| HIV/AIDS | 12106060000330 | Lamivudine |
| HIV/AIDS | 12106060002020 | Lamivudine |
| HIV/AIDS | 12108070000115 | Stavudine |
| HIV/AIDS | 12108070000120 | Stavudine |
| HIV/AIDS | 12108070000130 | Stavudine |
| HIV/AIDS | 12108070000140 | Stavudine |
| HIV/AIDS | 12108070002120 | Stavudine |
| HIV/AIDS | 12108085000110 | Zidovudine |
| HIV/AIDS | 12108085000330 | Zidovudine |
| HIV/AIDS | 12108085001210 | Zidovudine |
| HIV/AIDS | 12108085002020 | Zidovudine |
| HIV/AIDS | 12108570100305 | Tenofovir Disoproxil Fumarate |
| HIV/AIDS | 12108570100310 | Tenofovir Disoproxil Fumarate |
| HIV/AIDS | 12108570100315 | Tenofovir Disoproxil Fumarate |
| HIV/AIDS | 12108570100320 | Tenofovir Disoproxil Fumarate |
| HIV/AIDS | 12108570102920 | Tenofovir Disoproxil Fumarate |
| HIV/AIDS | 12109020200320 | Delavirdine Mesylate |
| HIV/AIDS | 12109020200330 | Delavirdine Mesylate |
| HIV/AIDS | 12109025000320 | Doravirine |
| HIV/AIDS | 12109030000110 | Efavirenz |
| HIV/AIDS | 12109030000140 | Efavirenz |
| HIV/AIDS | 12109030000330 | Efavirenz |
| HIV/AIDS | 12109035000310 | Etravirine |
| HIV/AIDS | 12109035000320 | Etravirine |
| HIV/AIDS | 12109035000340 | Etravirine |
| HIV/AIDS | 12109050000320 | Nevirapine |
| HIV/AIDS | 12109050001820 | Nevirapine |
| HIV/AIDS | 12109050007510 | Nevirapine |
| HIV/AIDS | 12109050007520 | Nevirapine |
| HIV/AIDS | 12109080100320 | Rilpivirine HCl |
| HIV/AIDS | 12109530000320 | Cobicistat |
| HIV/AIDS | 12109902200340 | Abacavir Sulfate-Lamivudine |
| HIV/AIDS | 12109902220330 | Atazanavir Sulfate-Cobicistat |
| HIV/AIDS | 12109902260320 | Dolutegravir Sodium-Lamivudine |
| HIV/AIDS | 12109902270320 | Darunavir-Cobicistat |
| HIV/AIDS | 12109902280320 | Dolutegravir Sodium-Rilpivirine HCl |
| HIV/AIDS | 12109902290320 | Emtricitabine-Tenofovir Alafenamide Fumarate |
| HIV/AIDS | 12109902300308 | Emtricitabine-Tenofovir Disoproxil Fumarate |
| HIV/AIDS | 12109902300312 | Emtricitabine-Tenofovir Disoproxil Fumarate |
| HIV/AIDS | 12109902300316 | Emtricitabine-Tenofovir Disoproxil Fumarate |
| HIV/AIDS | 12109902300320 | Emtricitabine-Tenofovir Disoproxil Fumarate |
| HIV/AIDS | 12109902470330 | Lamivudine-Tenofovir Disoproxil Fumarate |
| HIV/AIDS | 12109902500320 | Lamivudine-Zidovudine |
| HIV/AIDS | 12109902550310 | Lopinavir-Ritonavir |
| HIV/AIDS | 12109902550320 | Lopinavir-Ritonavir |
| HIV/AIDS | 12109902552020 | Lopinavir-Ritonavir |
| HIV/AIDS | 12109903150320 | Abacavir-Dolutegravir-Lamivudine |
| HIV/AIDS | 12109903200320 | Abacavir Sulfate-Lamivudine-Zidovudine |
| HIV/AIDS | 12109903240330 | Bictegravir-Emtricitabine-Tenofovir Alafenamide Fumarate |
| HIV/AIDS | 12109903270320 | Doravirine-Lamivudine-Tenofovir Disoproxil Fumarate |
| HIV/AIDS | 12109903300320 | Efavirenz-Emtricitabine-Tenofovir Disoproxil Fumarate |
| HIV/AIDS | 12109903330330 | Efavirenz-Lamivudine-Tenofovir Disoproxil Fumarate |
| HIV/AIDS | 12109903330340 | Efavirenz-Lamivudine-Tenofovir Disoproxil Fumarate |
| HIV/AIDS | 12109903390320 | Emtricitabine-Rilpivirine-Tenofovir Alafenamide Fumarate |
| HIV/AIDS | 12109903400320 | Emtricitabine-Rilpivirine-Tenofovir Disoproxil Fumarate |
| HIV/AIDS | 12109904200320 | Darunavir-Cobicistat-Emtricitabine-Tenofovir Alafenamide |
| HIV/AIDS | 12109904290315 | Elvitegravir-Cobicistat-Emtricitabine-Tenofovir Alafenamide |
| HIV/AIDS | 12109904300320 | Elvitegravir-Cobicistat-Emtricitabine-Tenofovir DF |
| Hemophilia and Related Dz | 30201010102015 | Desmopressin Acetate |
| Hemophilia and Related Dz | 84100040002025 | Tranexamic Acid |
| Hemophilia and Related Dz | 85100010002110 | Antihemophilic Factor (Human) |
| Hemophilia and Related Dz | 85100010002130 | Antihemophilic Factor (Human) |
| Hemophilia and Related Dz | 85100010002140 | Antihemophilic Factor (Human) |
| Hemophilia and Related Dz | 85100010002146 | Antihemophilic Factor (Human) |
| Hemophilia and Related Dz | 85100010006460 | Antihemophilic Factor (Human) |
| Hemophilia and Related Dz | 85100010006475 | Antihemophilic Factor (Human) |
| Hemophilia and Related Dz | 85100010202115 | Antihemophilic Factor (Recombinant) |
| Hemophilia and Related Dz | 85100010202120 | Antihemophilic Factor (Recombinant) |
| Hemophilia and Related Dz | 85100010202125 | Antihemophilic Factor (Recombinant) |
| Hemophilia and Related Dz | 85100010202130 | Antihemophilic Factor (Recombinant) |
| Hemophilia and Related Dz | 85100010202135 | Antihemophilic Factor (Recombinant) |
| Hemophilia and Related Dz | 85100010202140 | Antihemophilic Factor (Recombinant) |
| Hemophilia and Related Dz | 85100010202145 | Antihemophilic Factor (Recombinant) |
| Hemophilia and Related Dz | 85100010202150 | Antihemophilic Factor (Recombinant) |
| Hemophilia and Related Dz | 85100010202155 | Antihemophilic Factor (Recombinant) |
| Hemophilia and Related Dz | 85100010202160 | Antihemophilic Factor (Recombinant) |
| Hemophilia and Related Dz | 85100010202170 | Antihemophilic Factor (Recombinant) |
| Hemophilia and Related Dz | 85100010206420 | Antihemophilic Factor (Recombinant) |
| Hemophilia and Related Dz | 85100010206430 | Antihemophilic Factor (Recombinant) |
| Hemophilia and Related Dz | 85100010206440 | Antihemophilic Factor (Recombinant) |
| Hemophilia and Related Dz | 85100010206450 | Antihemophilic Factor (Recombinant) |
| Hemophilia and Related Dz | 85100010206460 | Antihemophilic Factor (Recombinant) |
| Hemophilia and Related Dz | 85100010222120 | Antihemophilic Factor (Recomb B-Domain Deleted) (BDD-rFVIII) |
| Hemophilia and Related Dz | 85100010222130 | Antihemophilic Factor (Recomb B-Domain Deleted) (BDD-rFVIII) |
| Hemophilia and Related Dz | 85100010222140 | Antihemophilic Factor (Recomb B-Domain Deleted) (BDD-rFVIII) |
| Hemophilia and Related Dz | 85100010222160 | Antihemophilic Factor (Recomb B-Domain Deleted) (BDD-rFVIII) |
| Hemophilia and Related Dz | 85100010222165 | Antihemophilic Factor (Recomb B-Domain Deleted) (BDD-rFVIII) |
| Hemophilia and Related Dz | 85100010222170 | Antihemophilic Factor (Recomb B-Domain Deleted) (BDD-rFVIII) |
| Hemophilia and Related Dz | 85100010222180 | Antihemophilic Factor (Recomb B-Domain Deleted) (BDD-rFVIII) |
| Hemophilia and Related Dz | 85100010226420 | Antihemophilic Factor (Recomb B-Domain Deleted) (BDD-rFVIII) |
| Hemophilia and Related Dz | 85100010226430 | Antihemophilic Factor (Recomb B-Domain Deleted) (BDD-rFVIII) |
| Hemophilia and Related Dz | 85100010226440 | Antihemophilic Factor (Recomb B-Domain Deleted) (BDD-rFVIII) |
| Hemophilia and Related Dz | 85100010226460 | Antihemophilic Factor (Recomb B-Domain Deleted) (BDD-rFVIII) |
| Hemophilia and Related Dz | 85100010226465 | Antihemophilic Factor (Recomb B-Domain Deleted) (BDD-rFVIII) |
| Hemophilia and Related Dz | 85100010226470 | Antihemophilic Factor (Recomb B-Domain Deleted) (BDD-rFVIII) |
| Hemophilia and Related Dz | 85100010226480 | Antihemophilic Factor (Recomb B-Domain Deleted) (BDD-rFVIII) |
| Hemophilia and Related Dz | 85100010252120 | Antihemophilic Factor rAHF-PFM |
| Hemophilia and Related Dz | 85100010252130 | Antihemophilic Factor rAHF-PFM |
| Hemophilia and Related Dz | 85100010252140 | Antihemophilic Factor rAHF-PFM |
| Hemophilia and Related Dz | 85100010252150 | Antihemophilic Factor rAHF-PFM |
| Hemophilia and Related Dz | 85100010252170 | Antihemophilic Factor rAHF-PFM |
| Hemophilia and Related Dz | 85100010252180 | Antihemophilic Factor rAHF-PFM |
| Hemophilia and Related Dz | 85100010252185 | Antihemophilic Factor rAHF-PFM |
| Hemophilia and Related Dz | 85100010266420 | Antihemophilic Factor (Recombinant) Plasma/Albumin Free |
| Hemophilia and Related Dz | 85100010266430 | Antihemophilic Factor (Recombinant) Plasma/Albumin Free |
| Hemophilia and Related Dz | 85100010266440 | Antihemophilic Factor (Recombinant) Plasma/Albumin Free |
| Hemophilia and Related Dz | 85100010266460 | Antihemophilic Factor (Recombinant) Plasma/Albumin Free |
| Hemophilia and Related Dz | 85100010266470 | Antihemophilic Factor (Recombinant) Plasma/Albumin Free |
| Hemophilia and Related Dz | 85100010302120 | Antihemophilic Factor (Recomb) Fc Fusion Protein (rFVIIIFc) |
| Hemophilia and Related Dz | 85100010302125 | Antihemophilic Factor (Recomb) Fc Fusion Protein (rFVIIIFc) |
| Hemophilia and Related Dz | 85100010302130 | Antihemophilic Factor (Recomb) Fc Fusion Protein (rFVIIIFc) |
| Hemophilia and Related Dz | 85100010302135 | Antihemophilic Factor (Recomb) Fc Fusion Protein (rFVIIIFc) |
| Hemophilia and Related Dz | 85100010302145 | Antihemophilic Factor (Recomb) Fc Fusion Protein (rFVIIIFc) |
| Hemophilia and Related Dz | 85100010302155 | Antihemophilic Factor (Recomb) Fc Fusion Protein (rFVIIIFc) |
| Hemophilia and Related Dz | 85100010302165 | Antihemophilic Factor (Recomb) Fc Fusion Protein (rFVIIIFc) |
| Hemophilia and Related Dz | 85100010302170 | Antihemophilic Factor (Recomb) Fc Fusion Protein (rFVIIIFc) |
| Hemophilia and Related Dz | 85100010302175 | Antihemophilic Factor (Recomb) Fc Fusion Protein (rFVIIIFc) |
| Hemophilia and Related Dz | 85100010302180 | Antihemophilic Factor (Recomb) Fc Fusion Protein (rFVIIIFc) |
| Hemophilia and Related Dz | 85100010352130 | Antihemophilic Factor (Recombinant) Glycopegylated-exei |
| Hemophilia and Related Dz | 85100010352140 | Antihemophilic Factor (Recombinant) Glycopegylated-exei |
| Hemophilia and Related Dz | 85100010352145 | Antihemophilic Factor (Recombinant) Glycopegylated-exei |
| Hemophilia and Related Dz | 85100010352150 | Antihemophilic Factor (Recombinant) Glycopegylated-exei |
| Hemophilia and Related Dz | 85100010352160 | Antihemophilic Factor (Recombinant) Glycopegylated-exei |
| Hemophilia and Related Dz | 85100010402120 | Antihemophilic Factor (Recombinant) Pegylated |
| Hemophilia and Related Dz | 85100010402130 | Antihemophilic Factor (Recombinant) Pegylated |
| Hemophilia and Related Dz | 85100010402135 | Antihemophilic Factor (Recombinant) Pegylated |
| Hemophilia and Related Dz | 85100010402140 | Antihemophilic Factor (Recombinant) Pegylated |
| Hemophilia and Related Dz | 85100010402145 | Antihemophilic Factor (Recombinant) Pegylated |
| Hemophilia and Related Dz | 85100010402150 | Antihemophilic Factor (Recombinant) Pegylated |
| Hemophilia and Related Dz | 85100010402160 | Antihemophilic Factor (Recombinant) Pegylated |
| Hemophilia and Related Dz | 85100010412130 | Antihemophilic Factor (Recombinant) Pegylated-aucl |
| Hemophilia and Related Dz | 85100010412140 | Antihemophilic Factor (Recombinant) Pegylated-aucl |
| Hemophilia and Related Dz | 85100010412150 | Antihemophilic Factor (Recombinant) Pegylated-aucl |
| Hemophilia and Related Dz | 85100010412160 | Antihemophilic Factor (Recombinant) Pegylated-aucl |
| Hemophilia and Related Dz | 85100010502130 | Antihemophilic Factor (Recombinant Porcine) (rpFVIII) |
| Hemophilia and Related Dz | 85100010556420 | Antihemophilic Factor (Recombinant) Single Chain |
| Hemophilia and Related Dz | 85100010556430 | Antihemophilic Factor (Recombinant) Single Chain |
| Hemophilia and Related Dz | 85100010556440 | Antihemophilic Factor (Recombinant) Single Chain |
| Hemophilia and Related Dz | 85100010556445 | Antihemophilic Factor (Recombinant) Single Chain |
| Hemophilia and Related Dz | 85100010556450 | Antihemophilic Factor (Recombinant) Single Chain |
| Hemophilia and Related Dz | 85100010556455 | Antihemophilic Factor (Recombinant) Single Chain |
| Hemophilia and Related Dz | 85100010556460 | Antihemophilic Factor (Recombinant) Single Chain |
| Hemophilia and Related Dz | 85100015102122 | Antihemophilic Factor/von Willebrand Factor Complex (Human) |
| Hemophilia and Related Dz | 85100015102129 | Antihemophilic Factor/von Willebrand Factor Complex (Human) |
| Hemophilia and Related Dz | 85100015102132 | Antihemophilic Factor/von Willebrand Factor Complex (Human) |
| Hemophilia and Related Dz | 85100015102139 | Antihemophilic Factor/von Willebrand Factor Complex (Human) |
| Hemophilia and Related Dz | 85100015102144 | Antihemophilic Factor/von Willebrand Factor Complex (Human) |
| Hemophilia and Related Dz | 85100015102160 | Antihemophilic Factor/von Willebrand Factor Complex (Human) |
| Hemophilia and Related Dz | 85100015102170 | Antihemophilic Factor/von Willebrand Factor Complex (Human) |
| Hemophilia and Related Dz | 85100015102180 | Antihemophilic Factor/von Willebrand Factor Complex (Human) |
| Hemophilia and Related Dz | 85100015102190 | Antihemophilic Factor/von Willebrand Factor Complex (Human) |
| Hemophilia and Related Dz | 85100015102193 | Antihemophilic Factor/von Willebrand Factor Complex (Human) |
| Hemophilia and Related Dz | 85100015106430 | Antihemophilic Factor/von Willebrand Factor Complex (Human) |
| Hemophilia and Related Dz | 85100015106440 | Antihemophilic Factor/von Willebrand Factor Complex (Human) |
| Hemophilia and Related Dz | 85100020002120 | Antiinhibitor Coagulant Complex |
| Hemophilia and Related Dz | 85100020002130 | Antiinhibitor Coagulant Complex |
| Hemophilia and Related Dz | 85100020002150 | Antiinhibitor Coagulant Complex |
| Hemophilia and Related Dz | 85100026202117 | Coagulation Factor VIIa (Recombinant) |
| Hemophilia and Related Dz | 85100026202126 | Coagulation Factor VIIa (Recombinant) |
| Hemophilia and Related Dz | 85100026202145 | Coagulation Factor VIIa (Recombinant) |
| Hemophilia and Related Dz | 85100026202160 | Coagulation Factor VIIa (Recombinant) |
| Hemophilia and Related Dz | 85100028002170 | Coagulation Factor IX |
| Hemophilia and Related Dz | 85100028002180 | Coagulation Factor IX |
| Hemophilia and Related Dz | 85100028002185 | Coagulation Factor IX |
| Hemophilia and Related Dz | 85100028202120 | Coagulation Factor IX (Recombinant) |
| Hemophilia and Related Dz | 85100028202130 | Coagulation Factor IX (Recombinant) |
| Hemophilia and Related Dz | 85100028202140 | Coagulation Factor IX (Recombinant) |
| Hemophilia and Related Dz | 85100028202145 | Coagulation Factor IX (Recombinant) |
| Hemophilia and Related Dz | 85100028202150 | Coagulation Factor IX (Recombinant) |
| Hemophilia and Related Dz | 85100028202160 | Coagulation Factor IX (Recombinant) |
| Hemophilia and Related Dz | 85100028206420 | Coagulation Factor IX (Recombinant) |
| Hemophilia and Related Dz | 85100028206430 | Coagulation Factor IX (Recombinant) |
| Hemophilia and Related Dz | 85100028206440 | Coagulation Factor IX (Recombinant) |
| Hemophilia and Related Dz | 85100028206450 | Coagulation Factor IX (Recombinant) |
| Hemophilia and Related Dz | 85100028206460 | Coagulation Factor IX (Recombinant) |
| Hemophilia and Related Dz | 85100028352110 | Coagulation Factor IX Recomb Albumin Fusion Protein (rIX-FP) |
| Hemophilia and Related Dz | 85100028352120 | Coagulation Factor IX Recomb Albumin Fusion Protein (rIX-FP) |
| Hemophilia and Related Dz | 85100028352130 | Coagulation Factor IX Recomb Albumin Fusion Protein (rIX-FP) |
| Hemophilia and Related Dz | 85100028352140 | Coagulation Factor IX Recomb Albumin Fusion Protein (rIX-FP) |
| Hemophilia and Related Dz | 85100028352150 | Coagulation Factor IX Recomb Albumin Fusion Protein (rIX-FP) |
| Hemophilia and Related Dz | 85100028402105 | Coagulation Factor IX (Recomb) Fc Fusion Protein (rFIXFc) |
| Hemophilia and Related Dz | 85100028402110 | Coagulation Factor IX (Recomb) Fc Fusion Protein (rFIXFc) |
| Hemophilia and Related Dz | 85100028402120 | Coagulation Factor IX (Recomb) Fc Fusion Protein (rFIXFc) |
| Hemophilia and Related Dz | 85100028402130 | Coagulation Factor IX (Recomb) Fc Fusion Protein (rFIXFc) |
| Hemophilia and Related Dz | 85100028402140 | Coagulation Factor IX (Recomb) Fc Fusion Protein (rFIXFc) |
| Hemophilia and Related Dz | 85100028402150 | Coagulation Factor IX (Recomb) Fc Fusion Protein (rFIXFc) |
| Hemophilia and Related Dz | 85100028452120 | Coagulation Factor IX (Recombinant) Glycopegylated |
| Hemophilia and Related Dz | 85100028452130 | Coagulation Factor IX (Recombinant) Glycopegylated |
| Hemophilia and Related Dz | 85100028452140 | Coagulation Factor IX (Recombinant) Glycopegylated |
| Hemophilia and Related Dz | 85100030002105 | Factor IX Complex |
| Hemophilia and Related Dz | 85100030002110 | Factor IX Complex |
| Hemophilia and Related Dz | 85100030002115 | Factor IX Complex |
| Hemophilia and Related Dz | 85100030002150 | Factor IX Complex |
| Hemophilia and Related Dz | 85100032102130 | Coagulation Factor XIII A-Subunit (Recombinant) |
| Hemophilia and Related Dz | 85100033006440 | Factor XIII Concentrate (Human) |
| Hemophilia and Related Dz | 85100070202120 | Von Willebrand Factor (Recombinant) |
| Hemophilia and Related Dz | 85100070202130 | Von Willebrand Factor (Recombinant) |
| Hemophilia and Related Dz | 85105030202010 | Emicizumab-kxwh |
| Hemophilia and Related Dz | 85105030202020 | Emicizumab-kxwh |
| Hemophilia and Related Dz | 85105030202030 | Emicizumab-kxwh |
| Hemophilia and Related Dz | 85105030202040 | Emicizumab-kxwh |
| Hepatitis (Non-C) | 12352015100320 | Adefovir Dipivoxil |
| Hepatitis (Non-C) | 12352030000320 | Entecavir |
| Hepatitis (Non-C) | 12352030000330 | Entecavir |
| Hepatitis (Non-C) | 12352030002020 | Entecavir |
| Hepatitis (Non-C) | 12352050000315 | Lamivudine (HBV) |
| Hepatitis (Non-C) | 12352050002010 | Lamivudine (HBV) |
| Hepatitis (Non-C) | 12352080000330 | Telbivudine |
| Hepatitis (Non-C) | 12352083200320 | Tenofovir Alafenamide Fumarate |
| Hepatitis (Non-C) | 19100010002050 | Hepatitis B Immune Globulin (Human) |
| Hepatitis C | 12353025100320 | Daclatasvir Dihydrochloride |
| Hepatitis C | 12353025100330 | Daclatasvir Dihydrochloride |
| Hepatitis C | 12353025100340 | Daclatasvir Dihydrochloride |
| Hepatitis C | 12353060106410 | Peginterferon alfa-2b |
| Hepatitis C | 12353060106416 | Peginterferon alfa-2b |
| Hepatitis C | 12353060106424 | Peginterferon alfa-2b |
| Hepatitis C | 12353060106430 | Peginterferon alfa-2b |
| Hepatitis C | 12353070000120 | Ribavirin (Hepatitis C) |
| Hepatitis C | 12353070000320 | Ribavirin (Hepatitis C) |
| Hepatitis C | 12353070000340 | Ribavirin (Hepatitis C) |
| Hepatitis C | 12353070000360 | Ribavirin (Hepatitis C) |
| Hepatitis C | 12353070002020 | Ribavirin (Hepatitis C) |
| Hepatitis C | 1235307000B715 | Ribavirin (Hepatitis C) |
| Hepatitis C | 1235307000B718 | Ribavirin (Hepatitis C) |
| Hepatitis C | 1235307000B720 | Ribavirin (Hepatitis C) |
| Hepatitis C | 1235307000B725 | Ribavirin (Hepatitis C) |
| Hepatitis C | 12353077100120 | Simeprevir Sodium |
| Hepatitis C | 12353080000310 | Sofosbuvir |
| Hepatitis C | 12353080000320 | Sofosbuvir |
| Hepatitis C | 12359902300320 | Elbasvir-Grazoprevir |
| Hepatitis C | 12359902350320 | Glecaprevir-Pibrentasvir |
| Hepatitis C | 12359902400310 | Ledipasvir-Sofosbuvir |
| Hepatitis C | 12359902400320 | Ledipasvir-Sofosbuvir |
| Hepatitis C | 12359902650330 | Sofosbuvir-Velpatasvir |
| Hepatitis C | 12359903600320 | Ombitasvir-Paritaprevir-Ritonavir |
| Hepatitis C | 12359903800330 | Sofosbuvir-Velpatasvir-Voxilaprevir |
| Hepatitis C | 12359904607530 | Ombitasvir-Paritaprevir-Ritonavir-Dasabuvir |
| Hepatitis C | 1235990460B720 | Ombitasvir-Paritaprevir-Ritonavir-Dasabuvir |
| Immunoglobulin Products | 19100005002200 | Cytomegalovirus Immune Globulin (Human) |
| Immunoglobulin Products | 19100020002200 | Immune Globulin (Human) IM |
| Immunoglobulin Products | 19100020102020 | Immune Globulin (Human) IV |
| Immunoglobulin Products | 19100020102030 | Immune Globulin (Human) IV |
| Immunoglobulin Products | 19100020102034 | Immune Globulin (Human) IV |
| Immunoglobulin Products | 19100020102038 | Immune Globulin (Human) IV |
| Immunoglobulin Products | 19100020102042 | Immune Globulin (Human) IV |
| Immunoglobulin Products | 19100020102044 | Immune Globulin (Human) IV |
| Immunoglobulin Products | 19100020102046 | Immune Globulin (Human) IV |
| Immunoglobulin Products | 19100020102063 | Immune Globulin (Human) IV |
| Immunoglobulin Products | 19100020102068 | Immune Globulin (Human) IV |
| Immunoglobulin Products | 19100020102072 | Immune Globulin (Human) IV |
| Immunoglobulin Products | 19100020102076 | Immune Globulin (Human) IV |
| Immunoglobulin Products | 19100020102080 | Immune Globulin (Human) IV |
| Immunoglobulin Products | 19100020102090 | Immune Globulin (Human) IV |
| Immunoglobulin Products | 19100020102120 | Immune Globulin (Human) IV |
| Immunoglobulin Products | 19100020102125 | Immune Globulin (Human) IV |
| Immunoglobulin Products | 19100020102130 | Immune Globulin (Human) IV |
| Immunoglobulin Products | 19100020102135 | Immune Globulin (Human) IV |
| Immunoglobulin Products | 19100020202050 | Immune Globulin (Human) Subcutaneous |
| Immunoglobulin Products | 19100020202054 | Immune Globulin (Human) Subcutaneous |
| Immunoglobulin Products | 19100020202058 | Immune Globulin (Human) Subcutaneous |
| Immunoglobulin Products | 19100020202062 | Immune Globulin (Human) Subcutaneous |
| Immunoglobulin Products | 19100020202065 | Immune Globulin (Human) Subcutaneous |
| Immunoglobulin Products | 19100020302060 | Immune Globulin (Human) IV or Subcutaneous |
| Immunoglobulin Products | 19100020302064 | Immune Globulin (Human) IV or Subcutaneous |
| Immunoglobulin Products | 19100020302068 | Immune Globulin (Human) IV or Subcutaneous |
| Immunoglobulin Products | 19100020302072 | Immune Globulin (Human) IV or Subcutaneous |
| Immunoglobulin Products | 19100020302076 | Immune Globulin (Human) IV or Subcutaneous |
| Immunoglobulin Products | 19100020302080 | Immune Globulin (Human) IV or Subcutaneous |
| Immunoglobulin Products | 19100020302084 | Immune Globulin (Human) IV or Subcutaneous |
| Immunoglobulin Products | 19100020572021 | Immune Globulin (Human)-hipp |
| Immunoglobulin Products | 19100020572025 | Immune Globulin (Human)-hipp |
| Immunoglobulin Products | 19100020572030 | Immune Globulin (Human)-hipp |
| Immunoglobulin Products | 19100020572035 | Immune Globulin (Human)-hipp |
| Immunoglobulin Products | 19100020572040 | Immune Globulin (Human)-hipp |
| Immunoglobulin Products | 19100020572055 | Immune Globulin (Human)-hipp |
| Immunoglobulin Products | 19100020602020 | Immune Globulin (Human)-ifas |
| Immunoglobulin Products | 19100020602025 | Immune Globulin (Human)-ifas |
| Immunoglobulin Products | 19100020602030 | Immune Globulin (Human)-ifas |
| Immunoglobulin Products | 19100020602035 | Immune Globulin (Human)-ifas |
| Immunoglobulin Products | 19100020602040 | Immune Globulin (Human)-ifas |
| Immunoglobulin Products | 19100020602045 | Immune Globulin (Human)-ifas |
| Immunoglobulin Products | 19100020642020 | Immune Globulin (Human)-klhw |
| Immunoglobulin Products | 19100020642025 | Immune Globulin (Human)-klhw |
| Immunoglobulin Products | 19100020642030 | Immune Globulin (Human)-klhw |
| Immunoglobulin Products | 19100020642040 | Immune Globulin (Human)-klhw |
| Immunoglobulin Products | 19100020802030 | Immune Globulin (Human)-slra |
| Immunoglobulin Products | 19100050002050 | Rho D Immune Globulin (Human) |
| Immunoglobulin Products | 19100050002055 | Rho D Immune Globulin (Human) |
| Immunoglobulin Products | 19100050002060 | Rho D Immune Globulin (Human) |
| Immunoglobulin Products | 19100050002065 | Rho D Immune Globulin (Human) |
| Immunoglobulin Products | 1910005000E520 | Rho D Immune Globulin (Human) |
| Immunoglobulin Products | 1910005000E540 | Rho D Immune Globulin (Human) |
| Immunoglobulin Products | 1910005000E550 | Rho D Immune Globulin (Human) |
| Immunoglobulin Products | 19990002356420 | Immune Globulin (Human)-Hyaluronidase (Human Recombinant) |
| Immunoglobulin Products | 19990002356425 | Immune Globulin (Human)-Hyaluronidase (Human Recombinant) |
| Immunoglobulin Products | 19990002356430 | Immune Globulin (Human)-Hyaluronidase (Human Recombinant) |
| Immunoglobulin Products | 19990002356440 | Immune Globulin (Human)-Hyaluronidase (Human Recombinant) |
| Immunoglobulin Products | 19990002356450 | Immune Globulin (Human)-Hyaluronidase (Human Recombinant) |
| Immunoglobulin Products | 21700060702020 | Interferon Gamma-1B |
| Immunoglobulin Products | 99402540102220 | Lymphocyte Immune Globulin,Anti-Thymocyte Globulin (Equine) |
| Immunoglobulin Products | 99402540302120 | Anti-Thymocyte Globulin (Rabbit), Lymphocyte Immune Globulin |
| Multiple Sclerosis | 6240003010E520 | Glatiramer Acetate |
| Multiple Sclerosis | 6240003010E540 | Glatiramer Acetate |
| Multiple Sclerosis | 6240101500B718 | Cladribine (Multiple Sclerosis) |
| Multiple Sclerosis | 6240101500B722 | Cladribine (Multiple Sclerosis) |
| Multiple Sclerosis | 6240101500B726 | Cladribine (Multiple Sclerosis) |
| Multiple Sclerosis | 6240101500B732 | Cladribine (Multiple Sclerosis) |
| Multiple Sclerosis | 6240101500B736 | Cladribine (Multiple Sclerosis) |
| Multiple Sclerosis | 6240101500B740 | Cladribine (Multiple Sclerosis) |
| Multiple Sclerosis | 6240101500B744 | Cladribine (Multiple Sclerosis) |
| Multiple Sclerosis | 62403060456420 | Interferon Beta-1a |
| Multiple Sclerosis | 6240306045D520 | Interferon Beta-1a |
| Multiple Sclerosis | 6240306045D540 | Interferon Beta-1a |
| Multiple Sclerosis | 6240306045D560 | Interferon Beta-1a |
| Multiple Sclerosis | 6240306045E520 | Interferon Beta-1a |
| Multiple Sclerosis | 6240306045E540 | Interferon Beta-1a |
| Multiple Sclerosis | 6240306045E560 | Interferon Beta-1a |
| Multiple Sclerosis | 6240306045F530 | Interferon Beta-1a |
| Multiple Sclerosis | 6240306045F830 | Interferon Beta-1a |
| Multiple Sclerosis | 62403060506420 | Interferon Beta-1b |
| Multiple Sclerosis | 6240307530D220 | Peginterferon Beta-1a |
| Multiple Sclerosis | 6240307530D250 | Peginterferon Beta-1a |
| Multiple Sclerosis | 6240307530E520 | Peginterferon Beta-1a |
| Multiple Sclerosis | 6240307530E550 | Peginterferon Beta-1a |
| Multiple Sclerosis | 62404070000320 | Teriflunomide |
| Multiple Sclerosis | 62404070000330 | Teriflunomide |
| Multiple Sclerosis | 62405010002020 | Alemtuzumab (MS) |
| Multiple Sclerosis | 6240502500E520 | Daclizumab (Multiple Sclerosis) |
| Multiple Sclerosis | 62405050001320 | Natalizumab |
| Multiple Sclerosis | 62405060002020 | Ocrelizumab |
| Multiple Sclerosis | 62405525006320 | Dimethyl Fumarate |
| Multiple Sclerosis | 62405525006520 | Dimethyl Fumarate |
| Multiple Sclerosis | 62405525006540 | Dimethyl Fumarate |
| Multiple Sclerosis | 62405530006520 | Diroximel Fumarate |
| Multiple Sclerosis | 62405530006540 | Diroximel Fumarate |
| Multiple Sclerosis | 62406030007420 | Dalfampridine |
| Multiple Sclerosis | 62407025100110 | Fingolimod HCl |
| Multiple Sclerosis | 62407025100120 | Fingolimod HCl |
| Multiple Sclerosis | 62407070200320 | Siponimod Fumarate |
| Multiple Sclerosis | 62407070200340 | Siponimod Fumarate |
| Multiple Sclerosis | 6240707020B720 | Siponimod Fumarate |
| Oncology - OAA | 17200010002200 | BCG Vaccine |
| Oncology - OAA | 21100009102005 | Bendamustine HCl |
| Oncology - OAA | 21100009102010 | Bendamustine HCl |
| Oncology - OAA | 21100009102030 | Bendamustine HCl |
| Oncology - OAA | 21100009102110 | Bendamustine HCl |
| Oncology - OAA | 21100009102120 | Bendamustine HCl |
| Oncology - OAA | 21100010002020 | Busulfan |
| Oncology - OAA | 21100015002030 | Carboplatin |
| Oncology - OAA | 21100015002035 | Carboplatin |
| Oncology - OAA | 21100015002040 | Carboplatin |
| Oncology - OAA | 21100015002045 | Carboplatin |
| Oncology - OAA | 21100020002020 | Cisplatin |
| Oncology - OAA | 21100020002025 | Cisplatin |
| Oncology - OAA | 21100020002030 | Cisplatin |
| Oncology - OAA | 21100020002110 | Cisplatin |
| Oncology - OAA | 21100028002025 | Oxaliplatin |
| Oncology - OAA | 21100028002030 | Oxaliplatin |
| Oncology - OAA | 21100028002120 | Oxaliplatin |
| Oncology - OAA | 21100028002130 | Oxaliplatin |
| Oncology - OAA | 21101020002120 | Cyclophosphamide |
| Oncology - OAA | 21101020002125 | Cyclophosphamide |
| Oncology - OAA | 21101020002130 | Cyclophosphamide |
| Oncology - OAA | 21101025002025 | Ifosfamide |
| Oncology - OAA | 21101025002030 | Ifosfamide |
| Oncology - OAA | 21101025002110 | Ifosfamide |
| Oncology - OAA | 21101025002130 | Ifosfamide |
| Oncology - OAA | 21101030102105 | Mechlorethamine HCl |
| Oncology - OAA | 21101040102110 | Melphalan HCl |
| Oncology - OAA | 21101040102115 | Melphalan HCl |
| Oncology - OAA | 21102010002105 | Carmustine |
| Oncology - OAA | 21102030002105 | Streptozocin |
| Oncology - OAA | 21104070002120 | Temozolomide |
| Oncology - OAA | 21107075002140 | Trabectedin |
| Oncology - OAA | 21200010102105 | Bleomycin Sulfate |
| Oncology - OAA | 21200010102115 | Bleomycin Sulfate |
| Oncology - OAA | 21200020002105 | Dactinomycin |
| Oncology - OAA | 21200030052210 | Daunorubicin Citrate Liposome |
| Oncology - OAA | 21200030102025 | Daunorubicin HCl |
| Oncology - OAA | 21200030102035 | Daunorubicin HCl |
| Oncology - OAA | 21200040102010 | Doxorubicin HCl |
| Oncology - OAA | 21200040102105 | Doxorubicin HCl |
| Oncology - OAA | 21200040102115 | Doxorubicin HCl |
| Oncology - OAA | 21200040402210 | Doxorubicin HCl Liposomal |
| Oncology - OAA | 21200042102030 | Epirubicin HCl |
| Oncology - OAA | 21200042102045 | Epirubicin HCl |
| Oncology - OAA | 21200045102025 | Idarubicin HCl |
| Oncology - OAA | 21200045102030 | Idarubicin HCl |
| Oncology - OAA | 21200045102035 | Idarubicin HCl |
| Oncology - OAA | 21200050002105 | Mitomycin |
| Oncology - OAA | 21200050002110 | Mitomycin |
| Oncology - OAA | 21200050002120 | Mitomycin |
| Oncology - OAA | 21200055001320 | Mitoxantrone HCl |
| Oncology - OAA | 21200055001325 | Mitoxantrone HCl |
| Oncology - OAA | 21200055001330 | Mitoxantrone HCl |
| Oncology - OAA | 21200080002020 | Valrubicin |
| Oncology - OAA | 21250010402125 | Asparaginase Erwinia Chrysanthemi |
| Oncology - OAA | 21250060002020 | Pegaspargase |
| Oncology - OAA | 21300003001920 | Azacitidine |
| Oncology - OAA | 21300007002015 | Cladribine |
| Oncology - OAA | 21300008002020 | Clofarabine |
| Oncology - OAA | 21300010002010 | Cytarabine |
| Oncology - OAA | 21300010002011 | Cytarabine |
| Oncology - OAA | 21300010002040 | Cytarabine |
| Oncology - OAA | 21300010301825 | Cytarabine Liposome |
| Oncology - OAA | 21300015002120 | Decitabine |
| Oncology - OAA | 21300020002105 | Floxuridine |
| Oncology - OAA | 21300025102020 | Fludarabine Phosphate |
| Oncology - OAA | 21300025102120 | Fludarabine Phosphate |
| Oncology - OAA | 21300030002020 | Fluorouracil |
| Oncology - OAA | 21300030002025 | Fluorouracil |
| Oncology - OAA | 21300030002030 | Fluorouracil |
| Oncology - OAA | 21300030002035 | Fluorouracil |
| Oncology - OAA | 21300034102020 | Gemcitabine HCl |
| Oncology - OAA | 21300034102040 | Gemcitabine HCl |
| Oncology - OAA | 21300034102060 | Gemcitabine HCl |
| Oncology - OAA | 21300034102073 | Gemcitabine HCl |
| Oncology - OAA | 21300034102077 | Gemcitabine HCl |
| Oncology - OAA | 21300034102080 | Gemcitabine HCl |
| Oncology - OAA | 21300034102083 | Gemcitabine HCl |
| Oncology - OAA | 21300034102110 | Gemcitabine HCl |
| Oncology - OAA | 21300034102140 | Gemcitabine HCl |
| Oncology - OAA | 21300034102160 | Gemcitabine HCl |
| Oncology - OAA | 21300034112020 | Gemcitabine HCl-Sodium Chloride |
| Oncology - OAA | 21300034112024 | Gemcitabine HCl-Sodium Chloride |
| Oncology - OAA | 21300034112028 | Gemcitabine HCl-Sodium Chloride |
| Oncology - OAA | 21300034112032 | Gemcitabine HCl-Sodium Chloride |
| Oncology - OAA | 21300034112036 | Gemcitabine HCl-Sodium Chloride |
| Oncology - OAA | 21300034112040 | Gemcitabine HCl-Sodium Chloride |
| Oncology - OAA | 21300034112044 | Gemcitabine HCl-Sodium Chloride |
| Oncology - OAA | 21300034112052 | Gemcitabine HCl-Sodium Chloride |
| Oncology - OAA | 21300034112056 | Gemcitabine HCl-Sodium Chloride |
| Oncology - OAA | 21300052002020 | Nelarabine |
| Oncology - OAA | 21300053102110 | Pemetrexed Disodium |
| Oncology - OAA | 21300053102120 | Pemetrexed Disodium |
| Oncology - OAA | 21300054002020 | Pralatrexate |
| Oncology - OAA | 21300054002025 | Pralatrexate |
| Oncology - OAA | 21335010102020 | Ziv-Aflibercept |
| Oncology - OAA | 21335010102030 | Ziv-Aflibercept |
| Oncology - OAA | 21335020002025 | Bevacizumab |
| Oncology - OAA | 21335020002030 | Bevacizumab |
| Oncology - OAA | 21335020202025 | Bevacizumab-awwb |
| Oncology - OAA | 21335020202030 | Bevacizumab-awwb |
| Oncology - OAA | 21335020302025 | Bevacizumab-bvzr |
| Oncology - OAA | 21335020302030 | Bevacizumab-bvzr |
| Oncology - OAA | 21335070002020 | Ramucirumab |
| Oncology - OAA | 21335070002040 | Ramucirumab |
| Oncology - OAA | 21352020002120 | Blinatumomab |
| Oncology - OAA | 21353010002040 | Alemtuzumab |
| Oncology - OAA | 21353015002015 | Atezolizumab |
| Oncology - OAA | 21353015002020 | Atezolizumab |
| Oncology - OAA | 21353020002020 | Avelumab |
| Oncology - OAA | 21353023402030 | Cemiplimab-rwlc |
| Oncology - OAA | 21353025002020 | Cetuximab |
| Oncology - OAA | 21353025002025 | Cetuximab |
| Oncology - OAA | 21353027002020 | Daratumumab |
| Oncology - OAA | 21353027002030 | Daratumumab |
| Oncology - OAA | 21353028002020 | Dinutuximab |
| Oncology - OAA | 21353029002020 | Durvalumab |
| Oncology - OAA | 21353029002030 | Durvalumab |
| Oncology - OAA | 21353030002120 | Elotuzumab |
| Oncology - OAA | 21353030002130 | Elotuzumab |
| Oncology - OAA | 21353032002020 | Ipilimumab |
| Oncology - OAA | 21353032002040 | Ipilimumab |
| Oncology - OAA | 21353036502120 | Moxetumomab Pasudotox-tdfk |
| Oncology - OAA | 21353038002020 | Necitumumab |
| Oncology - OAA | 21353041002020 | Nivolumab |
| Oncology - OAA | 21353041002030 | Nivolumab |
| Oncology - OAA | 21353041002050 | Nivolumab |
| Oncology - OAA | 21353043002025 | Obinutuzumab |
| Oncology - OAA | 21353045001320 | Ofatumumab |
| Oncology - OAA | 21353045001360 | Ofatumumab |
| Oncology - OAA | 21353047002010 | Olaratumab |
| Oncology - OAA | 21353047002020 | Olaratumab |
| Oncology - OAA | 21353050002025 | Panitumumab |
| Oncology - OAA | 21353050002035 | Panitumumab |
| Oncology - OAA | 21353053002030 | Pembrolizumab |
| Oncology - OAA | 21353053002120 | Pembrolizumab |
| Oncology - OAA | 21353054002020 | Pertuzumab |
| Oncology - OAA | 21353060002020 | Rituximab |
| Oncology - OAA | 21353060002040 | Rituximab |
| Oncology - OAA | 21353060102020 | Rituximab-abbs |
| Oncology - OAA | 21353060102040 | Rituximab-abbs |
| Oncology - OAA | 21353060602020 | Rituximab-pvvr |
| Oncology - OAA | 21353060602040 | Rituximab-pvvr |
| Oncology - OAA | 21353070002110 | Trastuzumab |
| Oncology - OAA | 21353070002120 | Trastuzumab |
| Oncology - OAA | 21353070142110 | Trastuzumab-anns |
| Oncology - OAA | 21353070142121 | Trastuzumab-anns |
| Oncology - OAA | 21353070302108 | Trastuzumab-dkst |
| Oncology - OAA | 21353070302120 | Trastuzumab-dkst |
| Oncology - OAA | 21353070652120 | Trastuzumab-qyyp |
| Oncology - OAA | 21355020202120 | Brentuximab Vedotin |
| Oncology - OAA | 21355026202120 | Enfortumab Vedotin-ejfv |
| Oncology - OAA | 21355026202130 | Enfortumab Vedotin-ejfv |
| Oncology - OAA | 21355030202117 | Gemtuzumab Ozogamicin |
| Oncology - OAA | 21355040202130 | Inotuzumab Ozogamicin |
| Oncology - OAA | 21355060302120 | Polatuzumab Vedotin-piiq |
| Oncology - OAA | 21355070302120 | Ado-Trastuzumab Emtansine |
| Oncology - OAA | 21355070302130 | Ado-Trastuzumab Emtansine |
| Oncology - OAA | 21355070552120 | Fam-Trastuzumab Deruxtecan-nxki |
| Oncology - OAA | 21358035406420 | Ibritumomab Tiuxetan for Yttrium-90 (Y-90) |
| Oncology - OAA | 21403530002024 | Fulvestrant |
| Oncology - OAA | 21405005102310 | Goserelin Acetate |
| Oncology - OAA | 21405005102330 | Goserelin Acetate |
| Oncology - OAA | 21405007106450 | Histrelin Acetate |
| Oncology - OAA | 21405010106410 | Leuprolide Acetate |
| Oncology - OAA | 21405010106415 | Leuprolide Acetate |
| Oncology - OAA | 21405010156430 | Leuprolide Acetate (3 Month) |
| Oncology - OAA | 21405010156432 | Leuprolide Acetate (3 Month) |
| Oncology - OAA | 21405010206430 | Leuprolide Acetate (4 Month) |
| Oncology - OAA | 21405010206435 | Leuprolide Acetate (4 Month) |
| Oncology - OAA | 21405010256445 | Leuprolide Acetate (6 Month) |
| Oncology - OAA | 21405010256450 | Leuprolide Acetate (6 Month) |
| Oncology - OAA | 21405050201920 | Triptorelin Pamoate |
| Oncology - OAA | 21405050201930 | Triptorelin Pamoate |
| Oncology - OAA | 21405050201940 | Triptorelin Pamoate |
| Oncology - OAA | 21405525102120 | Degarelix Acetate |
| Oncology - OAA | 21405525102130 | Degarelix Acetate |
| Oncology - OAA | 21500003002020 | Cabazitaxel |
| Oncology - OAA | 21500005001310 | Docetaxel |
| Oncology - OAA | 21500005001315 | Docetaxel |
| Oncology - OAA | 21500005001316 | Docetaxel |
| Oncology - OAA | 21500005001317 | Docetaxel |
| Oncology - OAA | 21500005001318 | Docetaxel |
| Oncology - OAA | 21500005001320 | Docetaxel |
| Oncology - OAA | 21500005001325 | Docetaxel |
| Oncology - OAA | 21500005002030 | Docetaxel |
| Oncology - OAA | 21500005002040 | Docetaxel |
| Oncology - OAA | 21500005002060 | Docetaxel |
| Oncology - OAA | 21500005002070 | Docetaxel |
| Oncology - OAA | 21500005002075 | Docetaxel |
| Oncology - OAA | 21500005002080 | Docetaxel |
| Oncology - OAA | 21500009202020 | Eribulin Mesylate |
| Oncology - OAA | 21500010002025 | Etoposide |
| Oncology - OAA | 21500010002030 | Etoposide |
| Oncology - OAA | 21500010002040 | Etoposide |
| Oncology - OAA | 21500010602120 | Etoposide Phosphate |
| Oncology - OAA | 21500011002120 | Ixabepilone |
| Oncology - OAA | 21500011002140 | Ixabepilone |
| Oncology - OAA | 21500012001325 | Paclitaxel |
| Oncology - OAA | 21500012001335 | Paclitaxel |
| Oncology - OAA | 21500012001340 | Paclitaxel |
| Oncology - OAA | 21500012001350 | Paclitaxel |
| Oncology - OAA | 21500012201920 | Paclitaxel Protein-Bound Particles |
| Oncology - OAA | 21500015002020 | Teniposide |
| Oncology - OAA | 21500020102005 | Vincristine Sulfate |
| Oncology - OAA | 21500020201820 | Vincristine Sulfate Liposome |
| Oncology - OAA | 21500030102020 | Vinblastine Sulfate |
| Oncology - OAA | 21500050802020 | Vinorelbine Tartrate |
| Oncology - OAA | 21500050802025 | Vinorelbine Tartrate |
| Oncology - OAA | 21531520002120 | Belinostat |
| Oncology - OAA | 21531560002120 | Romidepsin |
| Oncology - OAA | 21532570002020 | Temsirolimus |
| Oncology - OAA | 21536015002120 | Bortezomib |
| Oncology - OAA | 21536015002122 | Bortezomib |
| Oncology - OAA | 21536025002105 | Carfilzomib |
| Oncology - OAA | 21536025002110 | Carfilzomib |
| Oncology - OAA | 21536025002120 | Carfilzomib |
| Oncology - OAA | 21550040102025 | Irinotecan HCl |
| Oncology - OAA | 21550040102030 | Irinotecan HCl |
| Oncology - OAA | 21550040102035 | Irinotecan HCl |
| Oncology - OAA | 21550040102040 | Irinotecan HCl |
| Oncology - OAA | 21550040202220 | Irinotecan HCl Liposome |
| Oncology - OAA | 21550080102020 | Topotecan HCl |
| Oncology - OAA | 21550080102120 | Topotecan HCl |
| Oncology - OAA | 21574070401820 | Talimogene Laherparepvec |
| Oncology - OAA | 21574070401840 | Talimogene Laherparepvec |
| Oncology - OAA | 21600055002025 | Radium Ra 223 Dichloride |
| Oncology - OAA | 21651070001800 | Sipuleucel-T |
| Oncology - OAA | 21700008102020 | Arsenic Trioxide |
| Oncology - OAA | 21700008102030 | Arsenic Trioxide |
| Oncology - OAA | 21700013001930 | BCG Live Intravesical |
| Oncology - OAA | 21700013001940 | BCG Live Intravesical |
| Oncology - OAA | 21700020002105 | Dacarbazine |
| Oncology - OAA | 21700020002110 | Dacarbazine |
| Oncology - OAA | 21700040102120 | Omacetaxine Mepesuccinate |
| Oncology - OAA | 21700045002120 | Pentostatin |
| Oncology - OAA | 21700060202022 | Interferon Alfa-2B |
| Oncology - OAA | 21700060202030 | Interferon Alfa-2B |
| Oncology - OAA | 21700060202130 | Interferon Alfa-2B |
| Oncology - OAA | 21700060202135 | Interferon Alfa-2B |
| Oncology - OAA | 21700060202160 | Interferon Alfa-2B |
| Oncology - OAA | 21703020002120 | Aldesleukin |
| Oncology - OAA | 21707050002020 | Methoxsalen (Photopheresis) |
| Oncology - OAA | 21707070102140 | Porfimer Sodium |
| Oncology - OAA | 21756030002120 | Glucarpidase |
| Oncology - OAA | 21758010002120 | Amifostine |
| Oncology - OAA | 21758050002010 | Mesna |
| Oncology - OAA | 21765060002120 | Palifermin |
| Oncology - OAA | 21990002201930 | Daunorubicin-Cytarabine Liposome |
| Oncology - OAA | 21990002642020 | Rituximab-Hyaluronidase Human |
| Oncology - OAA | 21990002642040 | Rituximab-Hyaluronidase Human |
| Oncology - OAA | 21990002722020 | Trastuzumab-Hyaluronidase-oysk |
| Oncology - OAA | 30042060102006 | Pamidronate Disodium |
| Oncology - OAA | 30042060102009 | Pamidronate Disodium |
| Oncology - OAA | 30042060102012 | Pamidronate Disodium |
| Oncology - OAA | 30042060102120 | Pamidronate Disodium |
| Oncology - OAA | 30042060102140 | Pamidronate Disodium |
| Oncology - OAA | 30042090001320 | Zoledronic Acid |
| Oncology - OAA | 30042090002016 | Zoledronic Acid |
| Oncology - OAA | 30044530002030 | Denosumab |
| Oncology - OAA | 64200011102020 | Clonidine HCl (Analgesia) |
| Oncology - OAA | 64200011102040 | Clonidine HCl (Analgesia) |
| Oncology - OAA | 82502060002020 | Plerixafor |
| Oncology - OAA | 94200090102120 | Thyrotropin Alfa |
| Oncology - OAA | 98401008002010 | Citric Acid-Polysorbate 80 |
| Oncology - SAA | 12353060052020 | Peginterferon alfa-2a |
| Oncology - SAA | 12353060052030 | Peginterferon alfa-2a |
| Oncology - SAA | 12353060052040 | Peginterferon alfa-2a |
| Oncology - SAA | 21100005000110 | Altretamine |
| Oncology - SAA | 21100010000305 | Busulfan |
| Oncology - SAA | 21101020000105 | Cyclophosphamide |
| Oncology - SAA | 21101020000110 | Cyclophosphamide |
| Oncology - SAA | 21102020000105 | Lomustine |
| Oncology - SAA | 21102020000110 | Lomustine |
| Oncology - SAA | 21102020000115 | Lomustine |
| Oncology - SAA | 21102020000120 | Lomustine |
| Oncology - SAA | 21104070000110 | Temozolomide |
| Oncology - SAA | 21104070000120 | Temozolomide |
| Oncology - SAA | 21104070000140 | Temozolomide |
| Oncology - SAA | 21104070000143 | Temozolomide |
| Oncology - SAA | 21104070000147 | Temozolomide |
| Oncology - SAA | 21104070000150 | Temozolomide |
| Oncology - SAA | 21300005000320 | Capecitabine |
| Oncology - SAA | 21300005000350 | Capecitabine |
| Oncology - SAA | 21300040001830 | Mercaptopurine |
| Oncology - SAA | 21300050002080 | Methotrexate |
| Oncology - SAA | 21300060000305 | Thioguanine |
| Oncology - SAA | 21370030300320 | Glasdegib Maleate |
| Oncology - SAA | 21370030300335 | Glasdegib Maleate |
| Oncology - SAA | 21370060200120 | Sonidegib Phosphate |
| Oncology - SAA | 21370070000120 | Vismodegib |
| Oncology - SAA | 21402250000320 | Mitotane |
| Oncology - SAA | 21402410000320 | Apalutamide |
| Oncology - SAA | 21402425000320 | Darolutamide |
| Oncology - SAA | 21402430000120 | Enzalutamide |
| Oncology - SAA | 21402460000330 | Nilutamide |
| Oncology - SAA | 21402835000320 | Exemestane |
| Oncology - SAA | 21406010200310 | Abiraterone Acetate |
| Oncology - SAA | 21406010200320 | Abiraterone Acetate |
| Oncology - SAA | 21406010200330 | Abiraterone Acetate |
| Oncology - SAA | 21450080000110 | Pomalidomide |
| Oncology - SAA | 21450080000115 | Pomalidomide |
| Oncology - SAA | 21450080000120 | Pomalidomide |
| Oncology - SAA | 21450080000125 | Pomalidomide |
| Oncology - SAA | 21470080000320 | Venetoclax |
| Oncology - SAA | 21470080000340 | Venetoclax |
| Oncology - SAA | 21470080000360 | Venetoclax |
| Oncology - SAA | 2147008000B720 | Venetoclax |
| Oncology - SAA | 21500010000120 | Etoposide |
| Oncology - SAA | 21531010000305 | Abemaciclib |
| Oncology - SAA | 21531010000310 | Abemaciclib |
| Oncology - SAA | 21531010000315 | Abemaciclib |
| Oncology - SAA | 21531010000320 | Abemaciclib |
| Oncology - SAA | 21531060000120 | Palbociclib |
| Oncology - SAA | 21531060000130 | Palbociclib |
| Oncology - SAA | 21531060000140 | Palbociclib |
| Oncology - SAA | 2153107050B720 | Ribociclib Succinate |
| Oncology - SAA | 2153107050B740 | Ribociclib Succinate |
| Oncology - SAA | 2153107050B760 | Ribociclib Succinate |
| Oncology - SAA | 21531550100120 | Panobinostat Lactate |
| Oncology - SAA | 21531550100130 | Panobinostat Lactate |
| Oncology - SAA | 21531550100140 | Panobinostat Lactate |
| Oncology - SAA | 21531575000120 | Vorinostat |
| Oncology - SAA | 21532025100120 | Dabrafenib Mesylate |
| Oncology - SAA | 21532025100130 | Dabrafenib Mesylate |
| Oncology - SAA | 21532040000120 | Encorafenib |
| Oncology - SAA | 21532040000130 | Encorafenib |
| Oncology - SAA | 21532080000320 | Vemurafenib |
| Oncology - SAA | 21532225000320 | Erdafitinib |
| Oncology - SAA | 21532225000325 | Erdafitinib |
| Oncology - SAA | 21532225000330 | Erdafitinib |
| Oncology - SAA | 21532530000310 | Everolimus |
| Oncology - SAA | 21532530000320 | Everolimus |
| Oncology - SAA | 21532530000325 | Everolimus |
| Oncology - SAA | 21532530000330 | Everolimus |
| Oncology - SAA | 21532530007310 | Everolimus |
| Oncology - SAA | 21532530007320 | Everolimus |
| Oncology - SAA | 21532530007340 | Everolimus |
| Oncology - SAA | 21533030000130 | Midostaurin |
| Oncology - SAA | 21533050000320 | Regorafenib |
| Oncology - SAA | 21533060400320 | Sorafenib Tosylate |
| Oncology - SAA | 21533070300120 | Sunitinib Malate |
| Oncology - SAA | 21533070300130 | Sunitinib Malate |
| Oncology - SAA | 21533070300135 | Sunitinib Malate |
| Oncology - SAA | 21533070300140 | Sunitinib Malate |
| Oncology - SAA | 21533520000320 | Binimetinib |
| Oncology - SAA | 21533530200320 | Cobimetinib Fumarate |
| Oncology - SAA | 21533570100310 | Trametinib Dimethyl Sulfoxide |
| Oncology - SAA | 21533570100330 | Trametinib Dimethyl Sulfoxide |
| Oncology - SAA | 21533675200320 | Tazemetostat HBr |
| Oncology - SAA | 21533820000120 | Entrectinib |
| Oncology - SAA | 21533820000130 | Entrectinib |
| Oncology - SAA | 21533835200120 | Larotrectinib Sulfate |
| Oncology - SAA | 21533835200150 | Larotrectinib Sulfate |
| Oncology - SAA | 21533835202020 | Larotrectinib Sulfate |
| Oncology - SAA | 21534003000120 | Acalabrutinib |
| Oncology - SAA | 21534006100320 | Afatinib Dimaleate |
| Oncology - SAA | 21534006100330 | Afatinib Dimaleate |
| Oncology - SAA | 21534006100340 | Afatinib Dimaleate |
| Oncology - SAA | 21534007100120 | Alectinib HCl |
| Oncology - SAA | 21534008000320 | Axitinib |
| Oncology - SAA | 21534008000340 | Axitinib |
| Oncology - SAA | 21534009000320 | Avapritinib |
| Oncology - SAA | 21534009000330 | Avapritinib |
| Oncology - SAA | 21534009000340 | Avapritinib |
| Oncology - SAA | 21534010000330 | Brigatinib |
| Oncology - SAA | 21534010000350 | Brigatinib |
| Oncology - SAA | 21534010000365 | Brigatinib |
| Oncology - SAA | 2153401000B720 | Brigatinib |
| Oncology - SAA | 21534012000320 | Bosutinib |
| Oncology - SAA | 21534012000327 | Bosutinib |
| Oncology - SAA | 21534012000340 | Bosutinib |
| Oncology - SAA | 21534013100320 | Cabozantinib S-Malate |
| Oncology - SAA | 21534013100330 | Cabozantinib S-Malate |
| Oncology - SAA | 21534013100340 | Cabozantinib S-Malate |
| Oncology - SAA | 21534013106460 | Cabozantinib S-Malate |
| Oncology - SAA | 21534013106470 | Cabozantinib S-Malate |
| Oncology - SAA | 21534013106480 | Cabozantinib S-Malate |
| Oncology - SAA | 21534014000130 | Ceritinib |
| Oncology - SAA | 21534014000330 | Ceritinib |
| Oncology - SAA | 21534015000120 | Crizotinib |
| Oncology - SAA | 21534015000125 | Crizotinib |
| Oncology - SAA | 21534019000320 | Dacomitinib |
| Oncology - SAA | 21534019000330 | Dacomitinib |
| Oncology - SAA | 21534019000340 | Dacomitinib |
| Oncology - SAA | 21534020000320 | Dasatinib |
| Oncology - SAA | 21534020000340 | Dasatinib |
| Oncology - SAA | 21534020000350 | Dasatinib |
| Oncology - SAA | 21534020000354 | Dasatinib |
| Oncology - SAA | 21534020000360 | Dasatinib |
| Oncology - SAA | 21534020000380 | Dasatinib |
| Oncology - SAA | 21534025100320 | Erlotinib HCl |
| Oncology - SAA | 21534025100330 | Erlotinib HCl |
| Oncology - SAA | 21534025100360 | Erlotinib HCl |
| Oncology - SAA | 21534030000320 | Gefitinib |
| Oncology - SAA | 21534031200320 | Gilteritinib Fumarate |
| Oncology - SAA | 21534033000110 | Ibrutinib |
| Oncology - SAA | 21534033000120 | Ibrutinib |
| Oncology - SAA | 21534033000320 | Ibrutinib |
| Oncology - SAA | 21534033000330 | Ibrutinib |
| Oncology - SAA | 21534033000340 | Ibrutinib |
| Oncology - SAA | 21534033000350 | Ibrutinib |
| Oncology - SAA | 21534035100320 | Imatinib Mesylate |
| Oncology - SAA | 21534035100340 | Imatinib Mesylate |
| Oncology - SAA | 21534050100320 | Lapatinib Ditosylate |
| Oncology - SAA | 2153405420B210 | Lenvatinib Mesylate |
| Oncology - SAA | 2153405420B215 | Lenvatinib Mesylate |
| Oncology - SAA | 2153405420B220 | Lenvatinib Mesylate |
| Oncology - SAA | 2153405420B223 | Lenvatinib Mesylate |
| Oncology - SAA | 2153405420B230 | Lenvatinib Mesylate |
| Oncology - SAA | 2153405420B240 | Lenvatinib Mesylate |
| Oncology - SAA | 2153405420B244 | Lenvatinib Mesylate |
| Oncology - SAA | 2153405420B250 | Lenvatinib Mesylate |
| Oncology - SAA | 21534056000320 | Lorlatinib |
| Oncology - SAA | 21534056000330 | Lorlatinib |
| Oncology - SAA | 21534058100320 | Neratinib Maleate |
| Oncology - SAA | 21534060200110 | Nilotinib HCl |
| Oncology - SAA | 21534060200115 | Nilotinib HCl |
| Oncology - SAA | 21534060200125 | Nilotinib HCl |
| Oncology - SAA | 21534065200320 | Osimertinib Mesylate |
| Oncology - SAA | 21534065200330 | Osimertinib Mesylate |
| Oncology - SAA | 21534070100320 | Pazopanib HCl |
| Oncology - SAA | 21534073010120 | Pexidartinib HCl |
| Oncology - SAA | 21534075100320 | Ponatinib HCl |
| Oncology - SAA | 21534075100340 | Ponatinib HCl |
| Oncology - SAA | 21534085000320 | Vandetanib |
| Oncology - SAA | 21534085000340 | Vandetanib |
| Oncology - SAA | 21534095000120 | Zanubrutinib |
| Oncology - SAA | 21534940000320 | Ivosidenib |
| Oncology - SAA | 21535030200320 | Enasidenib Mesylate |
| Oncology - SAA | 21535030200340 | Enasidenib Mesylate |
| Oncology - SAA | 21535550200120 | Niraparib Tosylate |
| Oncology - SAA | 21535560000120 | Olaparib |
| Oncology - SAA | 21535560000330 | Olaparib |
| Oncology - SAA | 21535560000340 | Olaparib |
| Oncology - SAA | 21535570200320 | Rucaparib Camsylate |
| Oncology - SAA | 21535570200325 | Rucaparib Camsylate |
| Oncology - SAA | 21535570200330 | Rucaparib Camsylate |
| Oncology - SAA | 21535580400110 | Talazoparib Tosylate |
| Oncology - SAA | 21535580400120 | Talazoparib Tosylate |
| Oncology - SAA | 21536045100120 | Ixazomib Citrate |
| Oncology - SAA | 21536045100130 | Ixazomib Citrate |
| Oncology - SAA | 21536045100140 | Ixazomib Citrate |
| Oncology - SAA | 21537520200120 | Fedratinib HCl |
| Oncology - SAA | 21537560200310 | Ruxolitinib Phosphate |
| Oncology - SAA | 21537560200320 | Ruxolitinib Phosphate |
| Oncology - SAA | 21537560200325 | Ruxolitinib Phosphate |
| Oncology - SAA | 21537560200330 | Ruxolitinib Phosphate |
| Oncology - SAA | 21537560200335 | Ruxolitinib Phosphate |
| Oncology - SAA | 2153801000B720 | Alpelisib |
| Oncology - SAA | 2153801000B725 | Alpelisib |
| Oncology - SAA | 2153801000B730 | Alpelisib |
| Oncology - SAA | 21538030000120 | Duvelisib |
| Oncology - SAA | 21538030000130 | Duvelisib |
| Oncology - SAA | 21538040000320 | Idelalisib |
| Oncology - SAA | 21538040000330 | Idelalisib |
| Oncology - SAA | 21550080100120 | Topotecan HCl |
| Oncology - SAA | 21550080100140 | Topotecan HCl |
| Oncology - SAA | 2156006000B720 | Selinexor |
| Oncology - SAA | 2156006000B730 | Selinexor |
| Oncology - SAA | 2156006000B740 | Selinexor |
| Oncology - SAA | 2156006000B750 | Selinexor |
| Oncology - SAA | 21700050100105 | Procarbazine HCl |
| Oncology - SAA | 21700075206410 | Peginterferon alfa-2b (Antineoplastic) |
| Oncology - SAA | 21700075206420 | Peginterferon alfa-2b (Antineoplastic) |
| Oncology - SAA | 21700075206430 | Peginterferon alfa-2b (Antineoplastic) |
| Oncology - SAA | 21708080000110 | Tretinoin (Chemotherapy) |
| Oncology - SAA | 21708220000120 | Bexarotene |
| Oncology - SAA | 2199000260B730 | Ribociclib Succinate-Letrozole |
| Oncology - SAA | 2199000260B740 | Ribociclib Succinate-Letrozole |
| Oncology - SAA | 2199000260B760 | Ribociclib Succinate-Letrozole |
| Oncology - SAA | 21990002750320 | Trifluridine-Tipiracil |
| Oncology - SAA | 21990002750330 | Trifluridine-Tipiracil |
| Oncology - SAA | 90371050204030 | Mechlorethamine HCl (Topical) |
| Oncology - SAA | 90376220004020 | Bexarotene (Topical) |
| Oncology - SAA | 93000084203040 | Uridine Triacetate (Emergency Treatment) |
| Oncology - SAA | 99392070000120 | Thalidomide |
| Oncology - SAA | 99392070000130 | Thalidomide |
| Oncology - SAA | 99392070000135 | Thalidomide |
| Oncology - SAA | 99392070000140 | Thalidomide |
| Oncology - SAA | 99394050000110 | Lenalidomide |
| Oncology - SAA | 99394050000120 | Lenalidomide |
| Oncology - SAA | 99394050000130 | Lenalidomide |
| Oncology - SAA | 99394050000140 | Lenalidomide |
| Oncology - SAA | 99394050000145 | Lenalidomide |
| Oncology - SAA | 99394050000150 | Lenalidomide |
| Organ Transplant | 12200045000320 | Letermovir |
| Organ Transplant | 12200045000340 | Letermovir |
| Organ Transplant | 12200045002020 | Letermovir |
| Organ Transplant | 12200045002040 | Letermovir |
| Organ Transplant | 99402020000110 | Cyclosporine |
| Organ Transplant | 99402020000140 | Cyclosporine |
| Organ Transplant | 99402020002005 | Cyclosporine |
| Organ Transplant | 99402020002010 | Cyclosporine |
| Organ Transplant | 99402020300120 | Cyclosporine Modified (For Microemulsion) |
| Organ Transplant | 99402020300130 | Cyclosporine Modified (For Microemulsion) |
| Organ Transplant | 99402020300150 | Cyclosporine Modified (For Microemulsion) |
| Organ Transplant | 99402020302020 | Cyclosporine Modified (For Microemulsion) |
| Organ Transplant | 99403030100120 | Mycophenolate Mofetil |
| Organ Transplant | 99403030100330 | Mycophenolate Mofetil |
| Organ Transplant | 99403030101920 | Mycophenolate Mofetil |
| Organ Transplant | 99403030202120 | Mycophenolate Mofetil HCl |
| Organ Transplant | 99403030300620 | Mycophenolate Sodium |
| Organ Transplant | 99403030300630 | Mycophenolate Sodium |
| Organ Transplant | 99404035000320 | Everolimus (Immunosuppressant) |
| Organ Transplant | 99404035000325 | Everolimus (Immunosuppressant) |
| Organ Transplant | 99404035000330 | Everolimus (Immunosuppressant) |
| Organ Transplant | 99404035000335 | Everolimus (Immunosuppressant) |
| Organ Transplant | 99404070000310 | Sirolimus |
| Organ Transplant | 99404070000320 | Sirolimus |
| Organ Transplant | 99404070000330 | Sirolimus |
| Organ Transplant | 99404070002020 | Sirolimus |
| Organ Transplant | 99404080000105 | Tacrolimus |
| Organ Transplant | 99404080000110 | Tacrolimus |
| Organ Transplant | 99404080000120 | Tacrolimus |
| Organ Transplant | 99404080002010 | Tacrolimus |
| Organ Transplant | 99404080003010 | Tacrolimus |
| Organ Transplant | 99404080003030 | Tacrolimus |
| Organ Transplant | 99404080007005 | Tacrolimus |
| Organ Transplant | 99404080007010 | Tacrolimus |
| Organ Transplant | 99404080007020 | Tacrolimus |
| Organ Transplant | 99404080007510 | Tacrolimus |
| Organ Transplant | 99404080007515 | Tacrolimus |
| Organ Transplant | 99404080007520 | Tacrolimus |
| Organ Transplant | 99406010000305 | Azathioprine |
| Organ Transplant | 99406010000315 | Azathioprine |
| Organ Transplant | 99406010000325 | Azathioprine |
| Organ Transplant | 99408020002120 | Belatacept |
| Pulmonary Fibrosis | 45550060000120 | Pirfenidone |
| Pulmonary Fibrosis | 45550060000325 | Pirfenidone |
| Pulmonary Fibrosis | 45550060000345 | Pirfenidone |
| Pulmonary Fibrosis | 45554050200120 | Nintedanib Esylate |
| Pulmonary Fibrosis | 45554050200130 | Nintedanib Esylate |
| Pulmonary Hypertension | 40120070000310 | Selexipag |
| Pulmonary Hypertension | 40120070000315 | Selexipag |
| Pulmonary Hypertension | 40120070000320 | Selexipag |
| Pulmonary Hypertension | 40120070000325 | Selexipag |
| Pulmonary Hypertension | 40120070000330 | Selexipag |
| Pulmonary Hypertension | 40120070000335 | Selexipag |
| Pulmonary Hypertension | 40120070000340 | Selexipag |
| Pulmonary Hypertension | 40120070000345 | Selexipag |
| Pulmonary Hypertension | 4012007000B720 | Selexipag |
| Pulmonary Hypertension | 40134050000310 | Riociguat |
| Pulmonary Hypertension | 40134050000320 | Riociguat |
| Pulmonary Hypertension | 40134050000330 | Riociguat |
| Pulmonary Hypertension | 40134050000340 | Riociguat |
| Pulmonary Hypertension | 40134050000350 | Riociguat |
| Pulmonary Hypertension | 40143060100320 | Sildenafil Citrate (Pulmonary Hypertension) |
| Pulmonary Hypertension | 40143060101920 | Sildenafil Citrate (Pulmonary Hypertension) |
| Pulmonary Hypertension | 40143060102020 | Sildenafil Citrate (Pulmonary Hypertension) |
| Pulmonary Hypertension | 40143080000320 | Tadalafil (Pulmonary Hypertension) |
| Pulmonary Hypertension | 40160007000310 | Ambrisentan |
| Pulmonary Hypertension | 40160007000320 | Ambrisentan |
| Pulmonary Hypertension | 40160015000320 | Bosentan |
| Pulmonary Hypertension | 40160015000330 | Bosentan |
| Pulmonary Hypertension | 40160015007320 | Bosentan |
| Pulmonary Hypertension | 40160050000320 | Macitentan |
| Pulmonary Hypertension | 40170040102110 | Epoprostenol Sodium |
| Pulmonary Hypertension | 40170040102130 | Epoprostenol Sodium |
| Pulmonary Hypertension | 40170060002020 | Iloprost |
| Pulmonary Hypertension | 40170060002040 | Iloprost |
| Pulmonary Hypertension | 40170080002020 | Treprostinil |
| Pulmonary Hypertension | 40170080002050 | Treprostinil |
| Pulmonary Hypertension | 40170080002060 | Treprostinil |
| Pulmonary Hypertension | 40170080002070 | Treprostinil |
| Pulmonary Hypertension | 40170080002080 | Treprostinil |
| Pulmonary Hypertension | 40170080050410 | Treprostinil Diolamine |
| Pulmonary Hypertension | 40170080050415 | Treprostinil Diolamine |
| Pulmonary Hypertension | 40170080050420 | Treprostinil Diolamine |
| Pulmonary Hypertension | 40170080050425 | Treprostinil Diolamine |
| Pulmonary Hypertension | 40170080050435 | Treprostinil Diolamine |
| Respiratory Syncytial Virus | 19502060002015 | Palivizumab |
| Respiratory Syncytial Virus | 19502060002020 | Palivizumab |
