# Supplementary material for: Distribution of Patients at Risk for Complications Related to COVID-19 in the United States: Model Development Study
Source: JMIR Public Health Surveill. 2020 Jun 18;6(2):e19606. doi: 10.2196/19606 (PMC7304254; doi:10.2196/19606)
Supplement: Multimedia Appendix 2 [file publichealth_v6i2e19606_app2.docx]

Appendix B. Maintenance medications included in the COVID-19 risk calculation

| **Medispan Drug Group Description** | **GPI2** | **Risk Level** |
| --- | --- | --- |
| PENICILLINS | 01 | Normal |
| CEPHALOSPORINS | 02 | Normal |
| MACROLIDES | 03 | Normal |
| TETRACYCLINES | 04 | Normal |
| FLUOROQUINOLONES | 05 | Normal |
| AMINOGLYCOSIDES | 07 | Normal |
| SULFONAMIDES | 08 | Normal |
| ANTIMYCOBACTERIAL AGENTS | 09 | Normal |
| ANTIFUNGALS | 11 | Normal |
| ANTIVIRALS | 12 | Normal |
| ANTIMALARIALS | 13 | Normal |
| AMEBICIDES | 14 | Normal |
| ANTHELMINTICS | 15 | Normal |
| ANTI-INFECTIVE AGENTS - MISC. | 16 | Normal |
| PASSIVE IMMUNIZING AND TREATMENT AGENTS | 19 | Normal |
| ALLERGENIC EXTRACTS/BIOLOGICALS MISC | 20 | Normal |
| ANTINEOPLASTICS AND ADJUNCTIVE THERAPIES | 21 | Normal |
| CORTICOSTEROIDS | 22 | High |
| ANDROGENS-ANABOLIC | 23 | Normal |
| ANTIDIABETICS | 27 | High |
| THYROID AGENTS | 28 | Normal |
| OXYTOCICS | 29 | Normal |
| ENDOCRINE AND METABOLIC AGENTS - MISC. | 30 | High |
| CARDIOTONICS | 31 | High |
| ANTIANGINAL AGENTS | 32 | High |
| BETA BLOCKERS | 33 | High |
| CALCIUM CHANNEL BLOCKERS | 34 | High |
| ANTIARRHYTHMICS | 35 | High |
| ANTIHYPERTENSIVES | 36 | High |
| DIURETICS | 37 | High |
| VASOPRESSORS | 38 | High |
| ANTIHYPERLIPIDEMICS | 39 | High |
| CARDIOVASCULAR AGENTS - MISC. | 40 | High |
| ANTIHISTAMINES | 41 | Normal |
| NASAL AGENTS - SYSTEMIC AND TOPICAL | 42 | Normal |
| COUGH/COLD/ALLERGY | 43 | High |
| ANTIASTHMATIC AND BRONCHODILATOR AGENTS | 44 | High |
| RESPIRATORY AGENTS - MISC. | 45 | High |
| LAXATIVES | 46 | Normal |
| ANTIDIARRHEAL/PROBIOTIC AGENTS | 47 | Normal |
| ANTACIDS | 48 | Normal |
| ULCER DRUGS/ANTISPASMODICS/ANTICHOLINERGICS | 49 | Normal |
| ANTIEMETICS | 50 | Normal |
| DIGESTIVE AIDS | 51 | Normal |
| GASTROINTESTINAL AGENTS - MISC. | 52 | Normal |
| URINARY ANTI-INFECTIVES | 53 | Normal |
| GENITOURINARY AGENTS - MISCELLANEOUS | 56 | Normal |
| ADHD/ANTI-NARCOLEPSY/ANTI-OBESITY/ANOREXIANTS | 61 | Normal |
| ANALGESICS - NonNarcotic | 64 | Normal |
| ANALGESICS - OPIOID | 65 | Normal |
| ANALGESICS - ANTI-INFLAMMATORY | 66 | Normal |
| GENERAL ANESTHETICS | 70 | Normal |
| ANTICONVULSANTS | 72 | High |
| ANTIPARKINSON AND RELATED THERAPY AGENTS | 73 | High |
| NEUROMUSCULAR AGENTS | 74 | Normal |
| MUSCULOSKELETAL THERAPY AGENTS | 75 | Normal |
| ANTIMYASTHENIC/CHOLINERGIC AGENTS | 76 | High |
| HEMATOPOIETIC AGENTS | 82 | High |
| ANTICOAGULANTS | 83 | High |
| HEMOSTATICS | 84 | High |
| HEMATOLOGICAL AGENTS - MISC. | 85 | High |
| OTIC AGENTS | 87 | Normal |
| ANORECTAL AGENTS | 89 | Normal |
| ANTISEPTICS & DISINFECTANTS | 92 | High |
| ANTIDOTES AND SPECIFIC ANTAGONISTS | 93 | Normal |
| ALTERNATIVE MEDICINES | 95 | Normal |
| CHEMICALS | 96 | Normal |
| MISCELLANEOUS THERAPEUTIC CLASSES | 99 | Normal |
